# A practical guide to two-stage sporulation of *Pyricularia oryzae*: introducing a filter paper method and comparison with existing methods using strains from diverse grass hosts

**DOI:** 10.1186/s13007-025-01466-6

**Published:** 2025-11-18

**Authors:** Jie-Hao Ou, Kazuyuki Okazaki, Akito Kubota, Guan-Ying Huang, Yi-Nian Chen, Chi-Yu Chen

**Affiliations:** 1 Tohoku Agricultural Research Center, National Agriculture and Food Research Organization (NARO), Iwate, 0200123 Japan; 2 Department of Plant Pathology, National Chung Hsing University, Taichung, 40227 Taiwan; 3 Plant Pathology, Taiwan Agricultural Research Institute, Taichung, 413008 Taiwan

**Keywords:** *Pyricularia oryzae*, Conidia production, Sporulation methods, Comparative protocol, Poaceae, Virulence assay, Resistance screening, Grey leaf spot, Rice blast

## Abstract

****Background**:**

*Pyricularia oryzae* is a major fungal pathogen responsible for significant yield losses in rice. In recent years, diverse pathotypes have emerged as threats to other economically important grasses, including ryegrass, oats, wheat and foxtail millet. Research on host–pathogen interactions involving this species requires reliable spore production for inoculation. However, as a hemibiotrophic pathogen, *P. oryzae* often sporulates poorly on artificial media and typically requires specialized two-stage protocols for consistent spore production. Although several such methods have been developed, all were optimized for rice-derived strains and have not been systematically evaluated across strains from other hosts. There is also a practical need for a simple setup that allows advance preparation and frozen storage of spore stocks. Therefore, we developed a new two-stage filter paper method and compared it with four published protocols across 23 strains from 13 grass hosts.

****Results**:**

Comparative analysis showed strain specific differences in sporulation across methods, with no consistent link to phylogenetic lineage. The filter paper method reached an inoculum-competent concentration (defined here as $\ge 5\times 10^{4}$ spores/mL, suitable for routine spray inoculation) without any concentration step in 18 of 23 strains (78%), compared with TARI 16/23 (70%), IRRI 15/23 (65%), corn grain 14/23 (61%), and mycelial mat 3/23 (13%). Spores dried on filter paper were ready to use upon thawing and retained germination with no change in virulence after six months of storage at -40 $^\circ$C. Step by step protocols with illustrations are provided for all five methods, together with practical guidance for choosing a method based on laboratory conditions, available resources, and research objectives.

****Conclusions**:**

This study provides a comparative evaluation of two-stage sporulation methods for *Pyricularia* strains across diverse grass hosts. Among the five methods, the newly developed filter paper method shows the broadest applicability across strains while maintaining yields comparable to established protocols. It can be prepared for frozen storage and used directly after thawing, enabling advance preparation and bulk stocking of inoculum for virulence profiling, resistance breeding, and disease management. These findings are particularly relevant for laboratories in regions that are affected by, or at risk of, outbreaks caused by this pathogen.

## Introduction

*Pyricularia oryzae* (syn. *Magnaporthe oryzae*) is a widespread fungal pathogen that infects a wide range of grass (Poaceae) hosts, including rice (*Oryza sativa*), foxtail millet (*Setaria italica*), ryegrass (*Lolium* spp.), wheat (*Triticum aestivum*) and oats (*Avena sativa*) [5, 26]. The crop losses caused by this pathogen pose a serious threat to global food security by reducing the production of staple foods and decreasing the availability of forage for livestock. Its high genetic variability, partly driven by transposable elements, enables the rapid emergence of new virulent races capable of overcoming resistance genes introduced through breeding [20]. As a result, resistant cultivars frequently lose their effectiveness within just a few growing seasons, leading to a constant need for improved breeding strategies [32].

Standardized pathogenicity assays depend on a reliable source of asexual spores. However, *P. oryzae* vary not only in virulence but also in their capacity to sporulate under *in vitro* conditions [15], complicating the production of uniform spore batches essential for these assays.

Sporulation in *P. oryzae* is affected by several environmental and nutritional factors, including light quality (particularly blue and near-UV light), substrate pH, humidity, aeration, and the composition of the growth medium [2, 15, 30]. Some model strains, such as Guy11, can produce spores reliably under single-stage conditions. In these systems, the mycelium grows and forms spores directly on agar plates over a period of 7 to 10 days, provided that appropriate media and lighting are used [23]. However, many other strains do not sporulate consistently under such conditions. Moreover, in single-stage cultures, spore formation occurs simultaneously with vegetative growth, resulting in a population of spores that vary in age and developmental stage. Older spores, particularly those more than seven days old, tend to show reduced pathogenicity [6]. This heterogeneity limits the use of single-stage cultures when uniform spores are needed.

To overcome the limitations of single-stage systems, two-stage sporulation methods have been developed for *P. oryzae*. These methods separate the vegetative growth and sporulation phases, enabling spore formation within a defined period and yielding populations with more uniform maturity. Two-stage protocols can be categorized based on the physical state of the substrates used in each phase. Early studies established agar-to-agar systems, in which mycelia are transferred from nutrient-rich to nutrient-depleted agar, or subjected to physical disturbance such as surface scratching, to induce sporulation [10, 31]. Although simple, these protocols often require extensive handling and long incubation. To reduce culture time, later work introduced liquid to agar systems. In these, mycelia grow in liquid medium for three to four days, which is much shorter than the five to seven days needed on agar plates, before transfer to fresh agar for sporulation [4, 8, 12]. Other variations include liquid to solid systems, which produce compact mycelial mats by vacuum filtration, and solid to solid systems, which use low cost cereal grains as substrates in both stages. The latter provides a cost effective option while maintaining satisfactory spore yield [13, 18]. Notably, Latterell and Rossi [18] also described harvesting spores from solid substrates with volatile solvents such as Freon 113. After solvent evaporation, the dried spores remain viable under refrigeration or freezing for over twenty years. Despite occasional contamination, this method provided a practical approach for long-term storage of inoculum.

These two-stage protocols offer flexibility, allowing researchers to select methods that suit their objectives and laboratory resources. Consequently, they have become routine in rice blast resistance breeding programs at the Taiwan Agricultural Research Institute (TARI), the Japan International Research Center for Agricultural Sciences (JIRCAS) and the International Rice Research Institute (IRRI) [4, 14].

Despite these advances, gaps remain. Strains of *P. oryzae* infecting foxtail millet, ryegrass, wheat and oats have recently expanded their range and pose new threats to crop production [3, 29]. All existing sporulation protocols were developed using rice-associated strains, yet genomic studies reveal that strains from other hosts belong to distinct phylogenetic lineages with substantial genetic differences [11]. It is unclear whether methods optimized for rice-associated strains perform equally well with strains from other hosts. Moreover, most publications describe a single protocol without side by side comparisons under uniform conditions. Practical information on cost, equipment and required expertise is often missing, as are troubleshooting notes and step by step illustrations. These omissions can hinder adoption by new or less experienced laboratories.

To address these challenges, we evaluated five two-stage sporulation protocols using 23 strains of *Pyricularia* spp. collected from Poaceae hosts in both tropical (Taiwan) and temperate (Japan) regions. The objective was to assess the advantages and limitations of each protocol when applied to strains with diverse ecological and genetic backgrounds. We provide detailed, step-by-step instructions with illustrations, highlight common technical difficulties along with possible solutions, and present this work as a practical reference for researchers, plant breeders, and plant protection professionals working in regions affected by blast disease or preparing for its potential emergence.

## Materials and methods

### Fungal strains: cultivation, identification, and preservation

*P. oryzae* strains were obtained from rice and other Poaceae hosts in Taiwan and Japan. These included strains previously deposited in the NARO Genebank of Japan, as well as additional strains collected during the course of this study. To broaden the scope of comparison, we also included two *P. grisea* strains derived from southern crabgrass (*Digitaria ciliaris*), a species closely related to *P. oryzae* and historically regarded as conspecific [5]. All strains were preserved using the method described by Hayashi et al. [14] and stored at -40 $^\circ$C. Prior to sporulation assays, strains were revived and maintained on V8 agar medium (10% Campbell’s V8 vegetable juice, 0.02% CaCO_3_, 1.7% agar) to promote fresh mycelial growth.

Species identification was confirmed by amplifying ribosomal DNA (rDNA) regions, including the small subunit (SSU), internal transcribed spacer (ITS), large subunit (LSU), and intergenic spacer (IGS), using primers LR230 (5’-CCACAGCCAAGGGAACGGGCTTGG-3’) and LR220 (5’-CCCGCCGTTTACCCGCGCTTGG-3’), followed by sequencing on a Nanopore platform as described by Ou et al. [25]. The resulting sequences were compared against genome assembly entries in the International Nucleotide Sequence Database (INSD), and representative sequences were retrieved for phylogenetic analysis. All sequences were aligned using MAFFT [17]. Maximum likelihood analysis was performed with IQ-TREE 2 [22], and Bayesian inference was conducted using the cloud-based implementation of MrBayes 3.2 [27] available on NGPhylogeny.fr [19]. Phylogenetic trees were visualized using FigTree (https://tree.bio.ed.ac.uk/software/figtree/).

### Sporulation procedures

Five two-stage sporulation methods were evaluated, each involving transitions between different substrates or culture conditions. To improve clarity and maintain consistency throughout this study, we divided the cultivation process into three defined steps, with each step assigned a specific term used consistently throughout this study.

1. Pre-culture: Preparation of the initial inoculum for the first-stage culture.
2. First stage: The vegetative growth phase, during which the fungus develops mycelium that subsequently give rise to asexual reproductive structures (conidiophores and spores) during the second stage.
3. Second stage: The reproductive phase, triggered by environmental changes or external stimuli, during which conidiophores and spores are produced from the mycelia formed in the first stage.

Methods evaluated in this study are as follows:

i. Filter paper method: (solid-to-solid; this study; detailed procedures in Supplementary file 1)
ii. IRRI method: (agar-to-agar; [14]; detailed procedures in Supplementary file 2)
iii. Mycelial mat method: (liquid-to-solid; [18]; detailed procedures in Supplementary file 3)
iv. Corn grain method: (solid-to-solid; [18]; detailed procedures in Supplementary file 4)
v. TARI method: (liquid-to-agar; [4]; detailed procedures in Supplementary file 5)

All sporulation methods were performed in triplicate for each strain. Full protocols, including illustrations and media preparation, are provided in Supplementary files 1–6. The protocols in the Supplementary files are written for routine use and allow some flexibility in parameters such as incubation time and temperature. For routine work, room temperature typically ranges from 20 to 25 $^\circ$C. To ensure reproducibility, we fixed all key parameters in this study. Every step indicated as room temperature was conducted at 25 $^\circ$C. As a result, the procedures in the main text may differ slightly from the more general formats in the Supplementary files.

(i) Filter paper method (this study): Pre-culture: Fresh colonies revived from storage were inoculated at three evenly spaced points on a 9-cm yeast sucrose agar (YSA) plate and incubated at 25 $^\circ$C for 5 days.

First stage: The entire colony was transferred into 50 mL of yeast sucrose (YS) broth and briefly blended. Two sterile 7-cm filter papers were immersed in the resulting suspension, drained, and placed into 9-cm Petri dishes. The dishes were incubated at 25 $^\circ$C for 5 days.

Second stage: Approximately 20 mL of sterile water was added to each dish and allowed to soak for 1 h at 25 $^\circ$C. After discarding the water, an additional 20 mL was added and soaked overnight at 25 $^\circ$C. The water was then removed, and the filter papers were incubated for 4 days under the sporulation environment described in Supplementary file 6.

(ii) IRRI method [14] Pre-culture: Fresh colonies revived from storage were inoculated onto prune agar (PA) slants in test tubes and incubated at 25 $^\circ$C for 7 days.

First stage: Aerial mycelium was scraped from the pre-culture using a sterile inoculation loop, suspended in 10 mL of sterile water, and 2 mL of the suspension were spread onto a fresh 9-cm PA plate. The plate remained uncovered until excess moisture evaporated; it was then covered and incubated at 27 $^\circ$C for 7 days.

Second stage: Aerial mycelium was gently removed using a sterilized glass slide, and the plate was incubated for 4 days under the sporulation environment described in Supplementary file 6.

(iii) Mycelial mat method [18] Pre-culture: Fresh colonies revived from storage were cut into 1–2 mm blocks, and approximately 20 blocks were placed in 70 mL of yeast extract dextrose (YED) broth. Cultures were incubated at 25 $^\circ$C with shaking at 130 rpm for 5 days.

First stage: Thirty-five milliliters of the pre-culture were transferred into 200 mL of YED in a 500 mL flask and incubated under the same conditions for an additional 3 days.

Second stage: The culture was filtered through sterile filter paper using a 9-cm Büchner funnel to form a compact mycelial mat. The entire mat, along with the supporting filter paper, was then transferred to a 9-cm Petri dish and incubated for 4 days under the sporulation environment described in Supplementary file 6.

*Modifications*: This study adapted the original large-scale protocol described by Latterell and Rossi [18], which involved culturing 400 mL of YED in 1-L flasks and filtering through 12-cm Büchner funnels to obtain 12-cm diameter mats. Here, the culture volume was reduced by approximately 50%, using 200 mL of YED in 500 mL flasks and 9-cm funnels to produce 9-cm mats. Additionally, we omitted the step of removing the mat from the filter paper. Instead, the entire filter paper containing the mat was transferred directly to the Petri dish, which simplified handling. These modifications ensured consistency in scale across all evaluated methods, thereby enabling comparative analysis.

(iv) Corn grain method [18] Pre-culture: Fresh colonies revived from storage were cut into 1–2 mm blocks, and approximately 20 blocks were placed in 70 mL of YED broth. Cultures were incubated at 25 $^\circ$C with shaking at 130 rpm for 5 days.

First stage: One hundred grams of corn grains were boiled for 10 min, cooled for 30 min, and autoclaved in a 300 mL flask. After cooling, 10 mL of the pre-culture was added. The flask was gently shaken and incubated at 25 $^\circ$C for 5 days to allow colonization.

Second stage: The colonized grains were vigorously shaken to break up clumps, and 25 g portions were transferred to 9-cm Petri dishes and incubated for 4 days under the sporulation environment described in Supplementary file 6.

*Modifications*: This study adapted the original large-scale protocol described by Latterell and Rossi (1986). The starting quantity of corn grains was reduced from 200 g to 100 g to accommodate the increased number of samples in this study.

(v) TARI method [4] Pre-culture: Fresh colonies revived from storage were inoculated onto V8 agar and incubated at 25 $^\circ$C for 4 days.

First stage: Colonies were finely chopped and transferred into 50 mL of prune broth (PB) in 125 mL flasks, then incubated at 28 $^\circ$C with shaking at 130 rpm in complete darkness for 4 days.

Second stage: The liquid culture was briefly blended at low speed to produce a uniform suspension. Two milliliters were spread onto 9-cm oat flour agar (OFA) plates, allowed to air-dry, and then incubated for 4 days under the sporulation environment described in Supplementary file 6.

### Standardized sporulation environment

For all methods, each second-stage 9-cm plate was placed in a sterilized polypropylene tray (30 $\times$ 20 $\times$ 5 cm) lined with a sterilized, damp paper towel. Six plates were arranged flat in each tray. The tray was sealed with plastic wrap punctured with 20 holes (1–2 mm in diameter) using sterile tweezers. The trays were incubated in a chamber at 25 $^\circ$C for 4 days, illuminated continuously by a 15 W blacklight bulb positioned approximately 30 cm above the tray. For a full illustrated description of the incubation setup, see Supplementary file 6.

### Spore harvesting and efficiency evaluation

After sporulation, the contents of each 9 cm Petri dish were transferred into a 50 mL centrifuge tube containing 10 mL of sterile distilled water with 0.1 % Tween 20. Tubes were sealed and shaken vigorously for 10 s to dislodge spores. Spore concentration was estimated with a hemocytometer. When fewer than five spores appeared in a $0.1\,\mu \textrm{L}$ counting chamber (approximately below $5\times 10^4$ spores/mL), a line count method was applied: a $2\,\mu \textrm{L}$ aliquot was streaked on a glass slide and spores along the line were counted by microscopy to estimate concentration. Comparative data analysis was performed using the statsmodels package [28].

To enable clear comparison across methods and strains, spore concentrations were classified as:

- Low: less than $1\times 10^4$ spores/mL
- Suboptimal: $1\times 10^4$ to $<5\times 10^4$ spores/mL
- Moderate: $5\times 10^4$ to $<10\times 10^4$ spores/mL
- High: $10\times 10^4$ spores/mL or more

Following the threshold commonly used in pathogenicity assays [14], $5\times 10^4$ spores/mL was set as the minimum inoculum concentration. Strains in the moderate or high categories were considered to have achieved an inoculum-competent concentration.

### Cryopreservation of spores

*P. oryzae* strain p1 was used to evaluate the feasibility of spore cryopreservation. Spores produced using the filter paper method were dried overnight in a 40 $^\circ$C oven and stored at -40 $^\circ$C for six months.

### Spore germination rate

To evaluate the germination capacity of spores under different conditions, spores of strain p1 were obtained from five sporulation methods described in this study. In addition, spores produced using the filter paper method and cryopreserved at $-40\,^{\circ }$C for six months were included for comparison. Each spore suspension was filtered through sterile double-layer gauze to remove debris and adjusted to a final concentration of $5\times 10^4$ spores/mL. A 0.1 mL aliquot was plated onto V8 agar, and the plates were incubated at 25 °C for 24 h. Germination was assessed by examining at least 200 randomly selected spores per replicate under a microscope. A spore was considered germinated if the germ tube extended beyond the length of the spore body. Each treatment was performed in triplicate.

### Pathogenicity evaluation

Pathogenicity assays were conducted using spores of strain p1 on four plant species: rice (*Oryza sativa* cv. Koshihikari), wheat (*Triticum aestivum* cv. Kitakamikomugi), hybrid ryegrass (*Lolium* $\times$ *boucheanum* cv. High flora), and corn (*Zea mays* cv. SH2821). Plants were grown to the three- to five-leaf stage, placed horizontally on stainless steel trays, and their roots were wrapped in damp paper towels to maintain moisture.

The inoculation procedure was adapted from the leaf drop assay described by Molinari and Talbot [23], with spore suspensions replaced by agar disks coated with spores. The spores used for coating were identical to those tested in the germination assay, comprising freshly prepared spores from five sporulation methods and filter-method spores cryopreserved for six months. Each spore suspension was adjusted to $5\times 10^4$ spores/mL, and 2 mL was spread evenly onto the surface of water agar plates (20 g agar per litre of sterile distilled water). The plates were air dried in a clean bench until excess surface moisture had evaporated. Agar disks (6 mm diameter) were cut with a sterile punch and placed, spore side down, onto the uppermost fully expanded leaf, spaced at least 2 cm apart.

To ensure consistent contact between the disk and leaf surface, a custom 3D-printed plastic clip with embedded magnets (https://www.thingiverse.com/thing:7075210) was used to hold them against the tray. Immediately after inoculation, each tray was enclosed in a transparent plastic bag to maintain high humidity and incubated at 25 °C. Five days after inoculation, the inoculated leaves were scanned using a GT-S660 flatbed scanner (Seiko Epson Corporation, Japan), and lesion areas were manually annotated and measured using the VGG Image Annotator [7]. Lesion severity was scored on the 0–9 scale of the IRRI Standard Evaluation System [16]. Each inoculation used three independent spore batches with two inoculation points per batch, resulting in six inoculation points per spore source and plant combination.

## Results

### Strain sampling and DNA identification

We analyzed 23 *Pyricularia* spp. strains, including three strains collected from rice fields in Taiwan and 20 strains obtained from 13 Poaceae host species in Japan (see Supplementary file 7 for the complete strain list). The full-length nuclear ribosomal DNA (rDNA) region, including SSU, ITS, LSU, and IGS, was successfully amplified and sequenced for all strains. The resulting sequences have been deposited in the International Nucleotide Sequence Database (INSD) via the DNA Data Bank of Japan (DDBJ) [21].

Phylogenetic analysis based on these rDNA sequences placed 21 strains within the *P. oryzae* clade. These strains were further assigned to five well-supported lineages: Eleusine lineage #1, Eleusine lineage #2, Setaria lineage, Oryza lineage, and the Lolium and Triticum lineage, following the classification system proposed by Gladieux et al. [11]. The remaining two strains, both isolated from *Digitaria ciliaris*, were placed in the *P. grisea* clade.

### Evaluation of sporulation efficiency across methods and strains

All five two-stage sporulation methods supported production of *Pyricularia* spp. spores but their ability to reach inoculum-competent concentrations ($\ge 5\times 10^4$ spores/mL) varied. The filter paper method reached the threshold in 18 of 23 strains (78.3%), the TARI method in 16 strains (69.6%), the IRRI method in 15 strains (65.2%), and the corn grain method in 14 strains (60.9%), whereas the mycelial mat method did so in only 3 strains (13.0%) (Fig. 2). ANOVA showed significant effects of method and strain ($p<0.01$) and a significant interaction ($p<0.01$). Post hoc Tukey HSD comparisons indicated that the mycelial mat method produced significantly fewer spores than the TARI method ($p<0.1$) and no other pairwise differences were significant. In terms of lineage, strains in Setaria lineage produced more spores than strains in other lineages ($p<0.01$) while no differences were found among the remaining lineages. Among individual strains, p11 yielded more spores than any other strain ($p<0.01$). Four strains did not reach inoculum-competent concentrations in any method: p14 (*Eleusine coracana*, Eleusine lineage #1), p32 (*Digitaria ciliaris*, *P. grisea*), p4 (*Lolium multiflorum*, Lolium and Triticum lineages) and p4t (*Oryza sativa*, Oryza lineage).

### Germination rate and pathogenicity of spores from different sporulation methods

All spores from the five two-stage methods and cryopreserved spores germinated at $\ge 99\%$ (Table 1), indicating high viability across all spore sources and no loss of viability after six months of cryopreservation. A binomial generalized linear model comparing germination proportions among methods found no significant effect of method (likelihood-ratio $\chi ^{2}(5)=2.73$, $p=0.74$). Strain p1, isolated from ryegrass (*Lolium* sp.), was inoculated onto hybrid ryegrass (*Lolium* $\times$ *boucheanum*), wheat (*Triticum aestivum*), rice (*Oryza sativa*) and corn (*Zea mays*). Five days after inoculation, hybrid ryegrass developed scale 9 lesions (grayish lesions without distinct margins), wheat developed scale 7 lesions (broad, spindle shaped lesions with a brown margin), rice developed scale 1 lesions (tiny, pinpoint necrotic spots), and corn showed scale 0 (no visible symptoms). According to the IRRI Standard Evaluation System [16], scale values above 5 indicate susceptibility. Therefore, hybrid ryegrass and wheat were considered as susceptible to p1 infection. ANOVA on mean lesion area found no significant differences among spore sources, and each host exhibited the same severity score across all sources (Fig. 3).

**Fig. 1:**
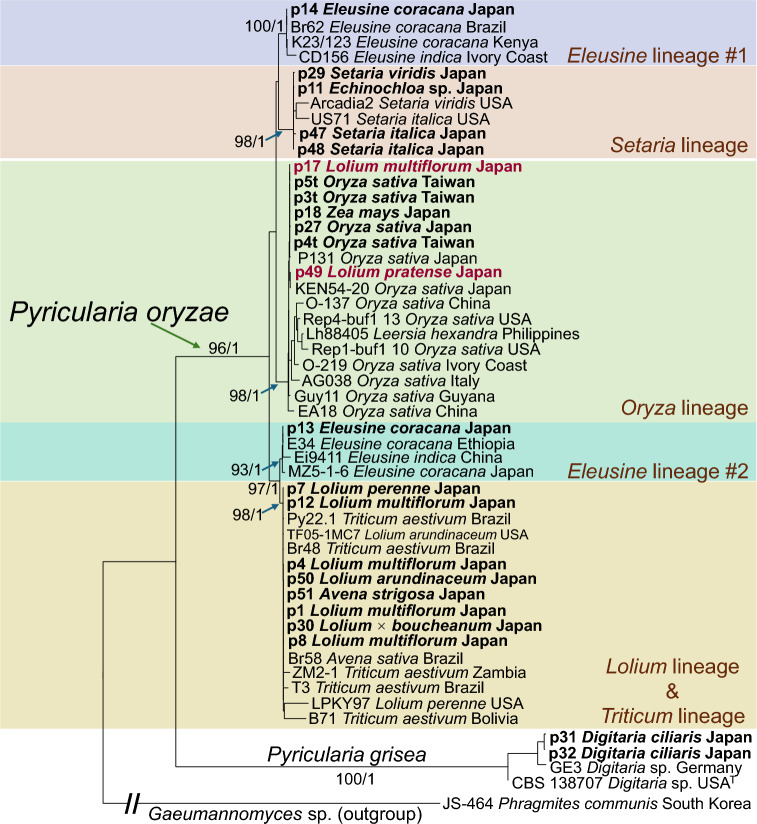
Maximum likelihood phylogenetic tree based on full-length rDNA sequences (SSU, ITS, LSU, IGS). Tip labels include strain name, isolation source, and locality. Bootstrap support (BS) and posterior probability (PP) values (BS/PP) are shown for nodes with BS $\ge$ 90 and PP $\ge$ 0.95. Lineage designations follow Gladieux et al. [11]. Holotype-derived sequences are marked with superscript “T”. Strains analyzed in this study are shown in bold. Strains p17 and p49, which were isolated from *Lolium* spp., were grouped within the Oryza lineage and are shown in red

**Fig. 2:**
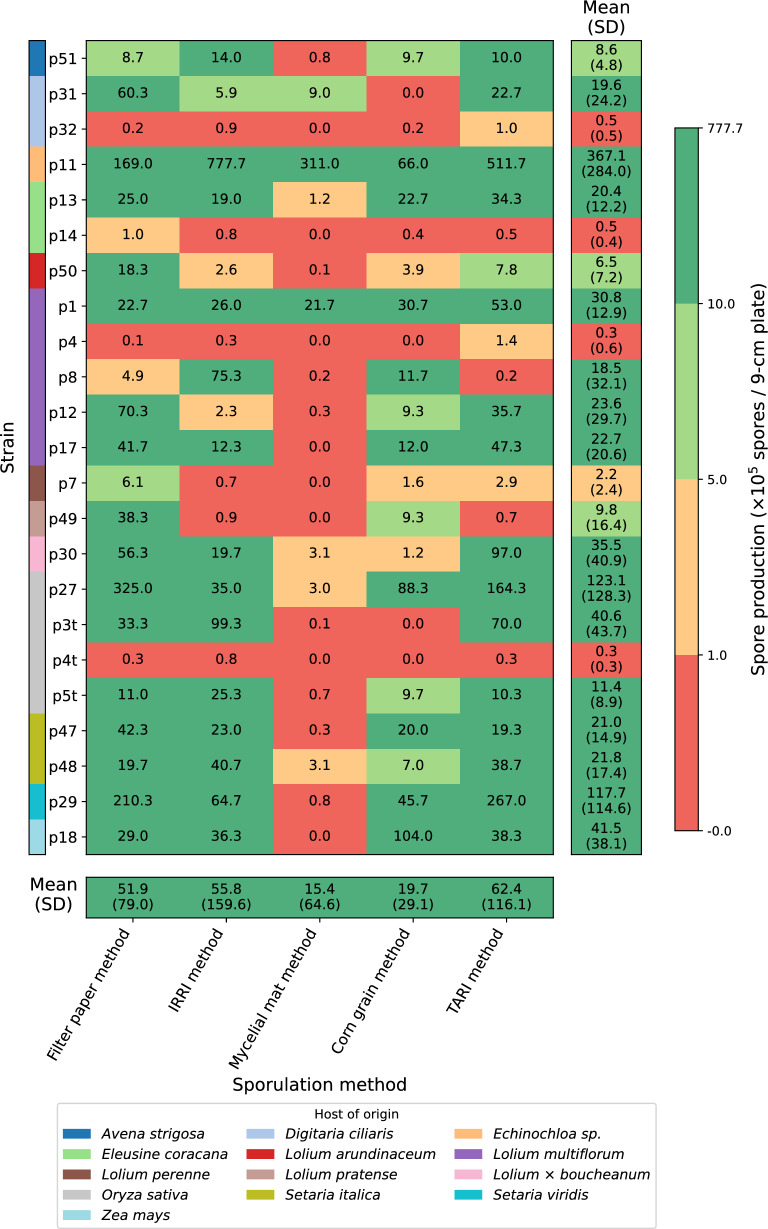
Comparison of sporulation efficiency across five two-stage protocols and *Pyricularia* spp. strains. Each cell shows the mean of three replicates and the number of spores harvested per 9 cm plate (units: $\times 10^{5}$ spores/plate). With the 10 mL wash per plate used in this study, these values are equivalent to $\times 10^{4}$ spores/mL in the final suspension. Cells are classified and color-coded into four categories: low (red; $<1\times 10^{4}$ spores/mL), suboptimal (orange; 1 to $<5\times 10^{4}$ spores/mL), moderate (light green; 5 to $<10\times 10^{4}$ spores/mL), and high (green; $\ge 10\times 10^{4}$ spores/mL). The left y-axis lists strain IDs. Host of origin is indicated by the color strip, explained in the legend, and detailed in Supplementary file 7. The right panel shows the mean (SD) across methods for each strain, and the bottom panel shows the mean (SD) across strains for each method

**Fig. 3:**
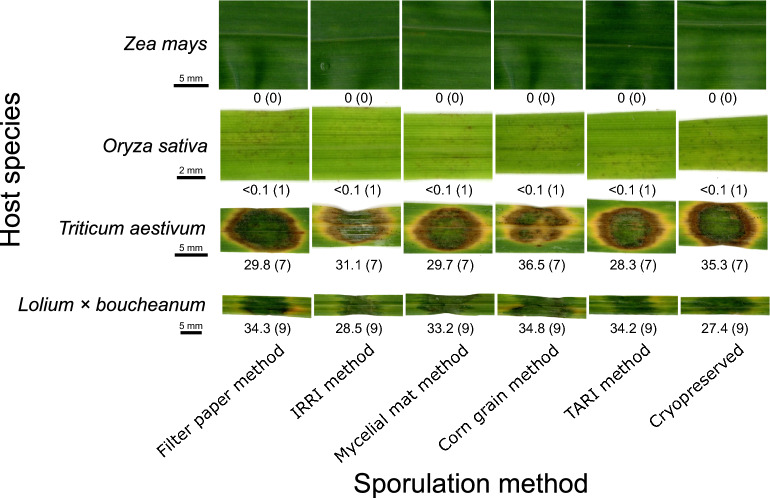
Pathogenicity of spores produced by five sporulation methods and from cryopreserved stocks. Leaf images were scanned five days after inoculation. For each image, the value below shows the mean lesion area (mm$^{2}$) with disease severity in parentheses. Lesion area is the mean of six inoculation points. Severity was scored for the dominant lesion type on a 0 to 9 scale according to the IRRI Standard Evaluation System [16]: 0 = no visible symptoms; 1 = pinpoint necrotic spots; 3 = small round brown necrotic spots (1–2 mm in diameter); 5 = narrow or slightly elliptical lesions; 7 = broad spindle shaped lesions with a brown margin; 9 = coalescing gray lesions with indistinct margins. For rice, scale 1 lesions had areas below 0.1 mm$^{2}$ and are reported as $<0.1$. Within each host row, a single scale bar applies to all images; the bar is placed beneath the scientific name shown on the y axis

**Table 1:** Spore germination by sporulation method (strain p1). Each entry shows germinated/total spores per replicate count ($\ge 200$ spores examined); the mean is calculated across three replicates

Method	Replicate 1	Replicate 2	Replicate 3	Mean germination (%)
Filter paper method	327/327 (100.00%)	215/215 (100.00%)	305/307 (99.35%)	99.78
IRRI method	230/230 (100.00%)	349/349 (100.00%)	258/259 (99.61%)	99.87
Mycelial mat method	287/287 (100.00%)	204/205 (99.51%)	289/289 (100.00%)	99.84
Corn grain method	267/267 (100.00%)	224/224 (100.00%)	338/338 (100.00%)	100.00
TARI method	276/277 (99.64%)	243/243 (100.00%)	301/301 (100.00%)	99.88
Cryopreserved	225/225 (100.00%)	312/312 (100.00%)	241/242 (99.59%)	99.86

## Discussion

This study analyzed a diverse collection of *Pyricularia* spp. strains obtained from a variety of grass hosts. Phylogenetic analysis based on the full-length rDNA region, particularly the IGS, revealed substantial genetic variation among the strains. When compared with the lineage framework proposed by Gladieux et al. [11], which is based on whole-genome data, the strains in this study were found to span multiple lineages. Notably, strains p17 and p49, although isolated from *Lolium* spp., clustered within the Oryza lineage instead of the expected Lolium lineage (as indicated in Fig. 1, marked in red). This unexpected placement may suggest a potential host shift, although further investigation of this hypothesis is beyond the scope of the present study.

These phylogenetically diverse strains were used to evaluate the performance of five two-stage sporulation protocols. While most methods supported spore production in the majority of strains, the mycelial mat method yielded inoculum-competent concentrations (i.e., $\ge 5 \times 10^4$ spores/mL) for pathogenicity assays in only three strains. This limitation, also noted in the original publication describing the method [18], suggests that it may be suitable only for a small subset of strains exhibiting high sporulation capacity.

Although strains in the Setaria lineage produced significantly more spores than those in other lineages, this difference likely reflects a few highly sporulating strains rather than a consistent phylogenetic pattern. Among the remaining *P. oryzae* strains, sporulation capacity did not correlate with lineage. For example, p1 and p4 were both classified in the Lolium lineage and isolated from Italian ryegrass (*Lolium multiflorum*), yet their sporulation profiles diverged markedly. A similar contrast was observed among three rice strains from Taiwan: p4t remained in the low category across all methods, whereas p5t consistently reached moderate to high levels of sporulation except under the mycelial mat protocol. The two *P. grisea* strains from *Digitaria ciliaris*, p31 and p32, also showed different outcomes. Under the filter paper and TARI methods, p31 reached the high category, while p32 remained in the low category for every method. These observations indicate that sporulation capacity depends more on individual strain characteristics than on host origin or phylogenetic lineage and may apply similarly to closely related species such as *P. grisea*.

### Selection of sporulation methods

Although sporulation efficiency varied across the five two-stage protocols, we standardized the inoculum to $5\times 10^{4}$ spores/mL for pathogenicity assays with strain p1. Under this condition, spores from all methods, including cryopreserved stocks, showed no significant differences in lesion area or severity on the hosts tested (Fig. 3). These results indicate that spores produced by any of the evaluated protocols are suitable for subsequent assays.

However, in germination rate tests, spores from the mycelial mat and corn grain protocols germinated more quickly and formed denser mycelial growth on V8 medium (data not shown), likely due to residual nutrients carried over from the substrate into the spore suspension. Therefore, we recommend that laboratories select a sporulation protocol based on cost, technical skill, personnel availability, and strain characteristics, and maintain the same protocol throughout a study to avoid unnecessary variation.

### Recommendations and precautions for experimental design

Below we summarize practical points for choosing and implementing a sporulation protocol.

i. Cost: The mycelial mat method incurred the highest consumable cost (approximately 0.2492 USD per plate) due to its high requirement for laboratory grade yeast extract. In contrast, the corn grain method was the most economical (approximately 0.1143 USD per plate) and proved effective for approximately 60% of strains. If necessary, costs can be further reduced by substituting imported reagents with locally available alternatives, such as food-grade yeast extract in place of laboratory grade yeast extract, or domestically produced vegetable juices instead of imported V8 juice.
ii. Storage: Fresh spores quickly lose viability and pathogenicity if not properly preserved, with significant declines reported after one week [6]. This poses a challenge for repeated or delayed inoculations. In contrast, spores that are dried immediately after harvesting can retain both viability and pathogenicity when stored at 4 °C or –20 °C [12, 18]. Most previously described protocols collect spores from moist substrates, followed by additional steps to separate and dry them. For instance, Latterell and Rossi [18] used organic solvents to extract and concentrate spores, while Guochang and Shuyuan [12] recovered spores from wash water by vacuum filtration.
  The filter paper method developed in this study simplifies this process and was shown to maintain both spore viability and pathogenicity after six months of storage at –40 °C. Because filter paper retains minimal moisture, spores can be dried within a few hours at 40 °C, preserving their viability without requiring solvent extraction, rinsing, or filtration. This reduces handling time and lowers the risk of contamination previously noted by Latterell and Rossi [18].
  For research projects requiring long-term spore preservation, the filter paper method offers a practical and efficient solution and is therefore the recommended approach.
iii. Growth duration: Liquid-based methods, such as the mycelial mat and TARI protocols, reduce the duration of the first-stage culture from 5–7 days to approximately 3–4 days. This feature is particularly advantageous for studies requiring rapid preparation. Accordingly, these methods are recommended when time constraints are a critical factor.
  However, the accelerated mycelial growth in liquid media can make it more difficult to determine the optimal timing for initiating the second stage. Transferring cultures either too early or too late may lead to reduced spore yields [24]. To support consistent results, we recommend performing preliminary growth curve assessments for each strain to identify the most suitable transition point and thereby maximize sporulation efficiency.
iv. Inoculation scale and space: Efficient use of laboratory space is essential for high-throughput breeding programs involving large-scale inoculation. The corn grain method is particularly suitable for such applications, as a single 300 mL bottle provides sufficient material for four second-stage cultures. The filter paper method requires slightly more space compared to the corn grain method, but remains practical. In contrast, liquid-based methods require bulky, temperature-controlled shaking incubators during the first stage, potentially limiting scalability in laboratories with restricted space.
v. Culture environment cleanliness: For laboratories with limited experience in microbiological techniques or operating under high contamination risk, especially in tropical regions, performing second-stage cultures on agar plates is often more suitable. Agar plates allow early detection of microbial contamination under a microscope. This is particularly true for the IRRI method, in which the transition from the first to the second stage does not involve a container change. This setup reduces the likelihood of introducing external contaminants and also lowers the risk of cross-contamination between different *P. oryzae* strains.
  Maintaining a clean culture environment throughout the second stage not only depends on the method itself but also on the use of appropriate sealing materials. Although parafilm is widely used in many laboratories to seal Petri dishes and other containers, we found that it deteriorates quickly under warm conditions. This often leads to small openings that compromise the seal. In this study, we recommend using 3 to 5 cm wide PVC plastic film, commonly employed for plant grafting. This material is more durable and helps maintain a stable culture environment throughout the incubation period.

### Limitations of this study

This study did not evaluate two important factors that may influence sporulation outcomes.

The first factor is the timing of transition from the first to the second stage of culture. Both immature and overly mature hyphae have been reported to exhibit reduced sporulation capacity [24]. The fixed culture durations applied in this study may not have been optimal for all strains. In addition, each method involved distinct media and culture conditions, which likely affected the growth curve. Therefore, poor sporulation observed in certain strain–method combinations may reflect suboptimal timing rather than limitations of the method itself. For example, in the mycelial mat method, strain p29 produced markedly more spores when the first-stage liquid culture duration was shortened from three days to 24 h, whereas strain p12 showed improved sporulation following an extended incubation period (data not shown). These observations suggest that the optimal timing for initiating the second stage is likely strain dependent and should be determined empirically.

The second factor is the nutrient composition of the culture media. Carbon and nitrogen availability are known to influence fungal development through nutrient repression mechanisms [1, 9]. Although the five sporulation methods tested here employed different media, the effects of nutrient content on sporulation were not systematically evaluated. In the TARI method, for instance, we observed that uneven distribution of insoluble oat particles in oat flour agar (OFA) plates led to inconsistent sporulation. Regions with dense oat content supported vigorous vegetative growth but reduced spore formation. A similar phenomenon was reported by Latterell and Rossi [18] for the corn grain method, where overcooked grains resulted in nutrient leakage from the endosperm, promoting mycelial growth while suppressing sporulation. These results indicate that sporulation in *Pyricularia* spp. is also influenced by nutrient repression, as has been described in other filamentous fungi. Future studies may benefit from optimizing nutrient formulations, particularly with respect to nutrient concentrations, to improve sporulation efficiency.

Finally, although four strains did not attain inoculum-competent concentrations under any of the tested methods, each strain produced at least a small number of spores under one or more protocols. When working with such low-sporulating strains and inoculation cannot be avoided, a practical workaround is to sequentially rinse multiple culture plates using the same suspension buffer. This allows spores to accumulate in a single suspension without increasing the final volume, although at the cost of additional labor.

## Conclusions

As climate change facilitates the emergence of *Pyricularia oryzae* in previously unaffected regions and on new host species, the need for effective and reproducible methods for spore production and preservation has become increasingly urgent. This is particularly relevant for strains derived from non-rice Poaceae hosts, many of which have not been extensively studied. Clear and accessible sporulation protocols are essential for establishing inoculation systems to support pathogenicity assays and resistance screening.

This study presents the first systematic comparison of two-stage sporulation protocols for *P. oryzae* strains that are often difficult to induce sporulation, including those collected from a broad range of Poaceae species encompassing major cereal and forage crops. We evaluated the strengths and limitations of several widely used methods under standardized conditions and introduced a newly developed filter paper method.

By providing detailed step-by-step procedures along with practical guidance, this work serves as a resource for laboratories aiming to establish reliable inoculum preparation protocols. It may be particularly useful for those working in regions where *P. oryzae* is newly emerging and where rapid development of experimental systems for epidemiological research, resistance evaluation, and breeding support is needed.

## Supplementary Information

Supplementary file 1. SuppFile1_Filter_paper_method.pdf – Filter paper method.

Supplementary file 2. SuppFile2_IRRI_method.pdf – IRRI method.

Supplementary file 3. SuppFile3_Mycelial_mat_method.pdf – Mycelial mat method.

Supplementary file 4. SuppFile4_Corn_grain_method.pdf – Corn grain method.

Supplementary file 5. SuppFile5_TARI_method.pdf – TARI method

Supplementary file 6. SuppFile6_Setup_of_Sporulation_Environment.pdf – Setup of the sporulation environment.

Supplementary file 7. SuppFile7_Strain_list.pdf – Strains used in this study and in the phylogenetic analysis.

## Data Availability

All sequence data generated in this study have been deposited in the INSD (International Nucleotide Sequence Databases) via DDBJ under accession numbers LC876988–LC877010.
