# Supplementary material for: A practical guide to two-stage sporulation of Pyricularia oryzae: introducing a filter paper method and comparison with existing methods using strains from diverse grass hosts
Source: Plant Methods. 2025 Nov 18;21:151. doi: 10.1186/s13007-025-01466-6 (PMC12625182; doi:10.1186/s13007-025-01466-6)
Supplement: Supplementary file 1 — SuppFile1_Filter_paper_method.pdf – Filter paper method. [file 13007_2025_1466_MOESM1_ESM.pdf]

FILTER PAPER METHOD

Supplementary Protocol 1: Filter paper method

Last edit July 13, 2025

PROTOCOL

Step 1 [Time required 5–7 days]

Pre-culture

1. Inoculate *P. oryzae* onto a 9 cm YSA plate at three evenly spaced points.
2. Incubate at 25°C in the dark for 5–7 days (Fig. S1).

> Depending on the strain, plates inoculated at three points typically reach 80 % coverage in 5–7 days. Use plates before full coverage, as older colonies may show reduced vigor and affect downstream steps.

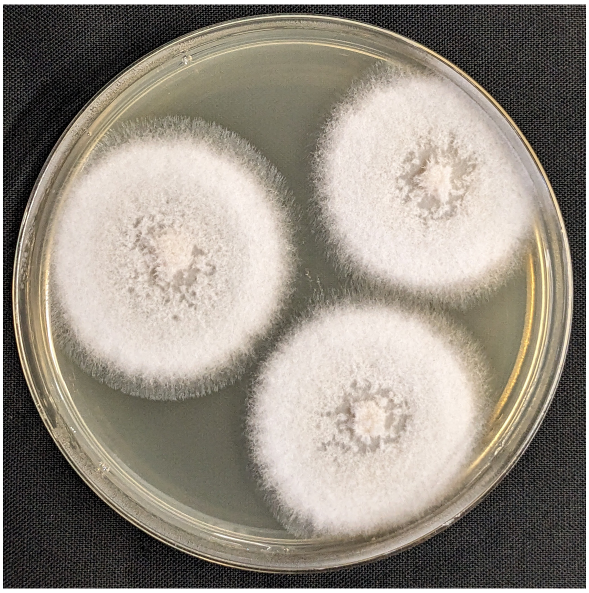

Figure S1: A typical colony of *P. oryzae* grown on YSA medium for 5–7 days.

Step 2 [Time required 5–7 days]

First stage

Option 1 (Preferred): Using Blender

1. Add 50 mL of YS medium to a sterile blender jar and autoclave.
2. Transfer colonies from pre-culture plates into the jar using sterile forceps (Fig. S2A, S2B).
3. Blend at highest speed for 10–15 s to obtain a mycelial suspension (Fig. S2C).
4. Dip two sterile 7 cm filter papers into the suspension; let excess liquid drip off, then place on fresh YS agar plates (Fig. S2D–S2G).
5. Incubate at 25°C for 5–7 days until mycelium covers filter paper (Fig. S2H).

MATERIALS

Checklist of required items

Equipment:

- Incubator or temperature-controlled space (for 25 °C incubation)
- Blender (Ensure the blender jar and blades are autoclavable)
- Autoclave (for media and tools sterilization)

Consumables:

- Yeast sucrose broth (YS)
  - Yeast extract (20 g/L)
  - Sucrose (50 g/L)
- Yeast sucrose agar (YSA)
  - YS plus agar (20 g/L)
- Filter paper (7 cm diameter)
- Petri dishes (plastic or glass, 9 cm)
- Sterile water

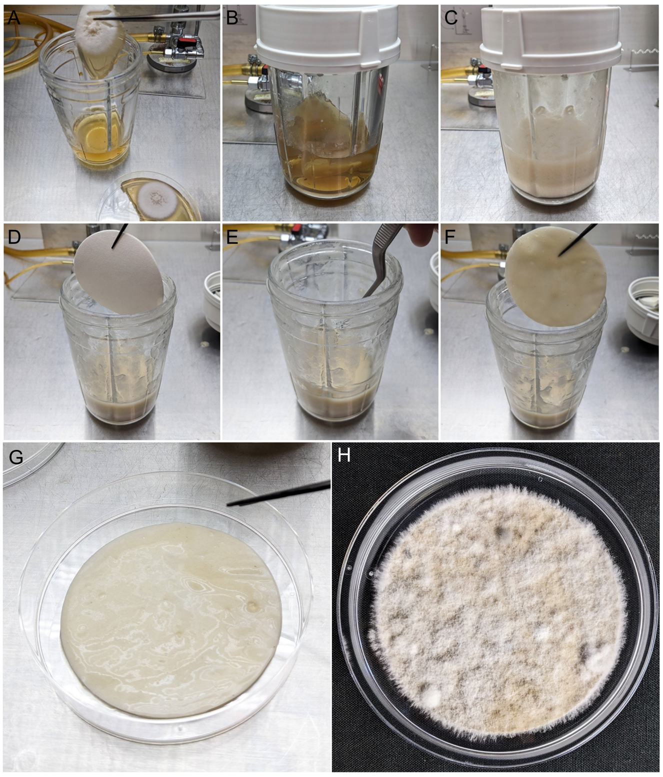

Figure S2: First-stage procedure. (A, B) Colonies in blender; (C) mycelial suspension; (D–G) dipping filter papers; (H) colonized filter paper after 5 days.

Option 2 (Alternative): Without Blender

1. Place two sterile 7 cm filter papers in a sterile 9 cm Petri dish (Fig. S3A).
2. Add 2 mL sterile YS medium (Fig. S3B).
3. Transfer $\geq$ 5 mycelial tips from colony margins onto each filter paper (Fig. S3C).
4. Incubate at 25°C for 5–7 days until coverage (Fig. S3D).

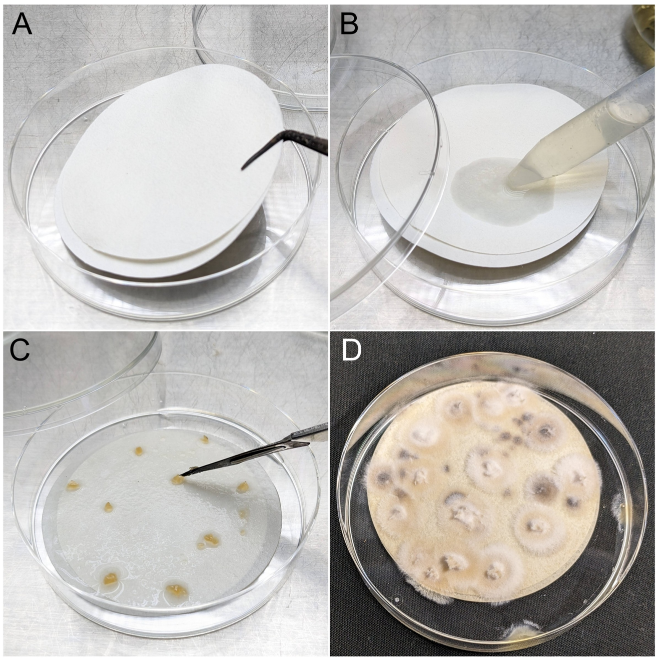

Figure S3: Alternative method without blender. (A) filter papers; (B) medium added; (C) mycelial tips; (D) growth after 5–7 days.

- For Option 2, use exactly 2 mL medium; excess liquid hinders growth.
- Tilt dish slightly during incubation to avoid water pooling on paper.

Step 3 [Time required 3–4 days]

Second stage

1. Add 20 mL sterile water to plates; soak 1 h at room temperature (Fig. S4A, S4B).
2. Decant water; add another 20 mL sterile water and soak $\geq$ 8 h at room temperature (Fig. S4C, S4D), then decant.
3. Incubate for 3–4 days at 25 °C in the specialized sporulation environment (see Supplementary Protocol 6), under near-UV (365 nm blacklight) or standard fluorescent lighting.

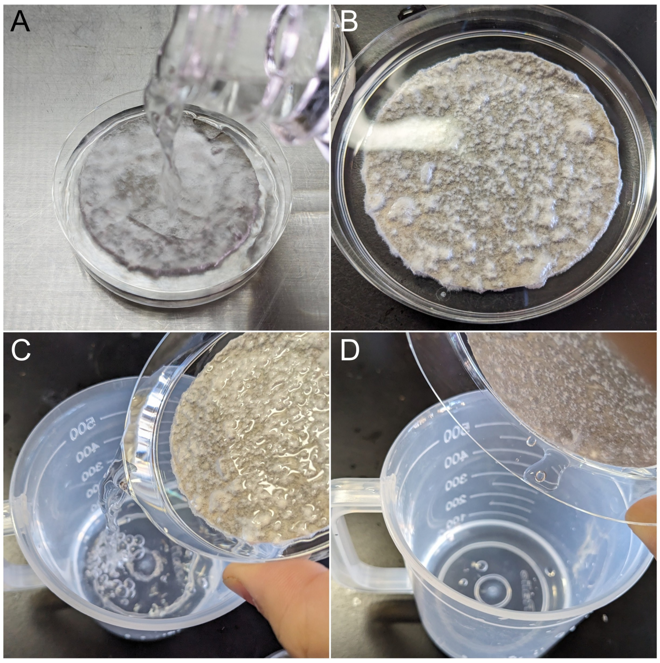

Figure S4: Second-stage procedure. (A) add water; (B) submerged colony; (C) decant after 1 h; (D) decant after overnight soak.

- Rinse removes excess nutrients and metabolites that can repress sporulation.
- Ensure removal of free water before incubation for efficient sporulation.

TROUBLESHOOTING

- **Low spore yield with little or no aerial mycelium:**
  - Transfer to the second stage before colonies reach the stationary phase; aging mycelium may lose the ability to produce spores efficiently.
  - Maintain high humidity ($\geq$97%) by adjusting the number of ventilation holes and keeping the paper towel beneath the dishes moist.
  - Keep the incubation temperature between 25–30 °C.
  - Check for contamination and confirm that the culture remains pure throughout both stages.
- **Low spore yield despite abundant aerial mycelium:**
  - Ensure that the incubation period of first stage is long enough for the culture to reach the late log phase, when sporulation is most efficient.
  - Make sure that excess nutrients are thoroughly removed before starting the second stage. For the filter paper method, increase the number of water rinses at the transition.
  - If excessive condensation or a noticeable ammonia odor is observed—both indicators of inadequate aeration—optimize airflow by increasing the ventilation openings in the plastic wrap or decreasing the plate density within the incubator.
- **High levels of contamination:**
  - Perform all pre-culture and first-stage steps under aseptic conditions. Avoid touching the rim of the blender jar directly; flame-sterilize all tools and vessel openings before and after use.
  - Ensure that all equipment and any water used for humidification are sterile.
- **Phenotypic changes in colonies:**
  - Use strains purified by mycelial tip or single-spore isolation. Avoid excessive subculturing, especially on nutrient-rich media such as potato dextrose agar.
  - For long-term storage, use filter paper preservation and revive strains from preserved stock only when necessary.
- **Many germinated or empty spores in harvested samples:**
  - Maintain proper humidity during the second stage to prevent condensation on aerial mycelium or conidiophores.
  - Keep incubation under seven days in the second stage to avoid premature germination or over-aging of spores.
  - In high-yield strains, some spores may form during the first stage and subsequently germinate or become empty during the second stage. To remove pre-existing spores, increase the number of rinses during the stage transition.

COST ANALYSIS

Consumable costs

- Yeast extract: \$0.09/g
- Sucrose: \$0.02/g
- Agar: \$0.17/g
- Filter paper: \$0.03/piece
- Petri dish: \$0.05/piece

Estimated consumable cost per second-stage plate

For each 9 cm plate used in the second stage, the estimated consumable costs are:

- **Yeast sucrose agar (YSA):** \$3.40/L $\times$ 0.002 L = \$0.0068
- **Yeast sucrose broth (YS):** \$2.80/L $\times$ 0.002 L = \$0.0056
- **Filter paper:** \$0.03/piece $\times$ 2 piece = \$0.06
- **Petri dishes:** \$0.05/piece $\times$ 1.1 piece = \$0.055

Total estimated cost per second-stage plate: \$0.1274

Notes:

- Cost of YSA and Petri dishes in the pre-culture stage is apportioned over ten first-stage plates (one pre-culture plate inoculates 10 first-stage plates).
- Only disposable consumables are included; reusable items (e.g. blender jar and blades) are excluded.
