# Supplementary material for: A practical guide to two-stage sporulation of Pyricularia oryzae: introducing a filter paper method and comparison with existing methods using strains from diverse grass hosts
Source: Plant Methods. 2025 Nov 18;21:151. doi: 10.1186/s13007-025-01466-6 (PMC12625182; doi:10.1186/s13007-025-01466-6)
Supplement: Supplementary file 2 — SuppFile2_IRRI_method.pdf – IRRI method. [file 13007_2025_1466_MOESM2_ESM.pdf]

IRRI METHOD

Supplementary Protocol 2: IRRI method (Hayashi et al., 2009)

Last edit July 13, 2025

PROTOCOL

Step 1 [Time required 7–10 days]

Pre-culture

1. Inoculate the *P. oryzae* strain onto a slant of PA medium in a glass test tube.
2. Incubate at 25°C for 7–10 days (Fig. S1).

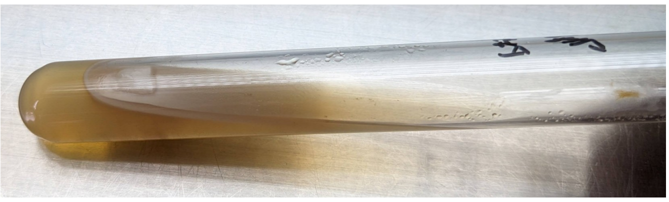

Figure S1: A typical colony of *P. oryzae* after 7 days of incubation on a PA slant.

- Use standard 50–100 mL glass test tubes containing 3–5 mL of medium.
- Use breathable stoppers to ensure proper fungal growth; cotton plugs are an alternative.
- Avoid excess free water in tubes, which can affect fungal growth.

Step 2 [Time required 7 days]

First stage

1. Use a sterile inoculation loop to gently scrape aerial mycelium from the surface of the PA slant (Fig. S2A).
2. Transfer the collected mycelium into a centrifuge tube containing 10 mL of sterile distilled water to prepare a suspension (Fig. S2B).
3. Using a sterile dropper, dispense 2 mL of the suspension onto a 9 cm PA plate (Fig. S2C).
4. Evenly spread the suspension across the medium surface using a sterile spreader, and allow excess moisture to evaporate briefly under a clean bench (Fig. S2D–E).
5. Incubate the plate at 27°C for 7 days.

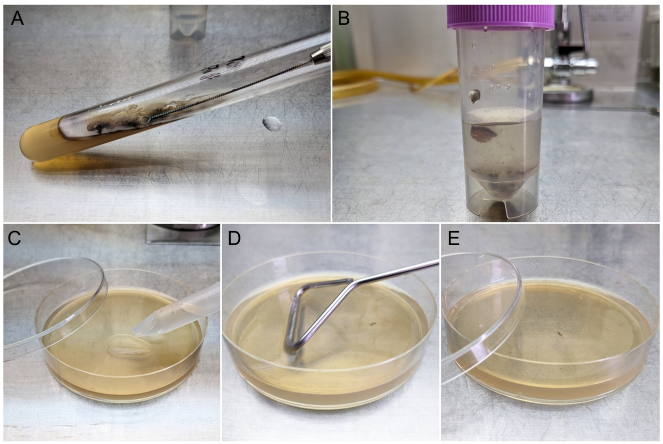

Figure S2: First-stage procedure of the IRRI method. (A) Scraping aerial mycelium from the slant. (B) Suspending mycelium in sterile water. (C) Dispensing suspension onto a PA plate. (D–E) Spreading the suspension and brief drying before incubation.

MATERIALS

Checklist of required items

Equipment:

- Incubator or temperature-controlled space (25 °C)
- Autoclave (for media and tool sterilization)
- Glass test tubes (50–100 mL) with breathable stoppers or cotton plugs
- Plastic centrifuge tubes (>10 mL)
- Pipettes or sterile droppers
- Triangle spreader
- Glass slide or autoclavable toothbrush

Consumables:

- **Prune Agar (PA):**
  - Prunes: 3 pieces (≈30 g)/L
  - Lactose: 5 g/L
  - Yeast extract: 1 g/L
  - Agar: 17 g/L
  - Adjust pH to 6.5
- Sterile water
- Petri dishes (9 cm, plastic or glass)

To prepare Prune Agar (PA): Boil prunes in about half the final volume of water for one hour. Crush the softened prunes, then filter out solids through a fine sieve. Adjust the filtrate to the final volume with water. Add lactose, yeast extract and agar, mix thoroughly. Adjust pH to 6.5 using NaOH before autoclaving to avoid agar degradation. If a pH meter is unavailable, 0.1 % $CaCO_3$ may be used to buffer the medium.

- Use cultures in early stationary phase, as older colonies lose sporulation ability.
- Mycelial fragments promote uniform coverage but require strict sterile handling.
- Alternatively, inoculate with multiple agar blocks (≥10 points) to reduce contamination risk.

Step 3 [Time required 3–4 days]

Second stage

1. Use a sterilized slide glass or soft toothbrush to gently remove aerial mycelium from the colony surface (Fig. S3A–B).
2. Incubate for 3–4 days at 25 °C in the specialized sporulation environment (see Supplementary Protocol 6), under near-UV (365 nm blacklight) or standard fluorescent lighting.

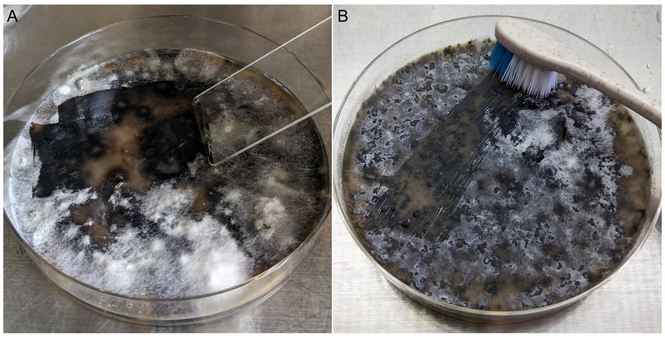

Figure S3: Second-stage procedure of the IRRI method. (A) Removing aerial mycelium with a sterilized slide glass. (B) Alternative removal using a soft toothbrush.

TROUBLESHOOTING

- **Low spore yield with little or no aerial mycelium:**
  - Transfer to the second stage before colonies reach the stationary phase; aging mycelium may lose the ability to produce spores efficiently.
  - Maintain high humidity ($\geq$97%) by adjusting the number of ventilation holes and keeping the paper towel beneath the dishes moist.
  - Keep the incubation temperature between 25–30 °C.
  - Check for contamination and confirm that the culture remains pure throughout both stages.
- **Low spore yield despite abundant aerial mycelium:**
  - Ensure that the incubation period of first stage is long enough for the culture to reach the late log phase, when sporulation is most efficient.
  - If excessive condensation or a noticeable ammonia odor is observed—both indicators of inadequate aeration—optimize airflow by increasing the ventilation openings in the plastic wrap or decreasing the plate density within the incubator.
- **High levels of contamination:**
  - Perform all pre-culture and first-stage steps under aseptic conditions. Avoid touching the rim of the glass tube directly; flame-sterilize all tools and vessel openings before and after use.
  - Ensure that all equipment and any water used for humidification are sterile.
- **Phenotypic changes in colonies:**
  - Use strains purified by mycelial tip or single-spore isolation. Avoid excessive subculturing, especially on nutrient-rich media such as potato dextrose agar.
  - For long-term storage, use filter paper preservation and revive strains from preserved stock only when necessary.
- **Many germinated or empty spores in harvested samples:**
  - Maintain proper humidity during the second stage to prevent condensation on aerial mycelium or conidiophores.
  - Keep incubation under seven days in the second stage to avoid premature germination or over-aging of spores.

COST ANALYSIS

Consumable costs

- Prune: $0.15/piece
- Lactose: $0.08/g
- Yeast extract: $0.09/g
- Agar: $0.17/g
- Petri dishes: $0.05/piece

Estimated consumable cost per second-stage plate

For each 9 cm plate in the second stage, the estimated consumable costs are:

- **Prune Agar (PA):** $3.83/L × 0.021 L = $0.08043
- **Petri dish:** $0.05/piece × 1 piece = $0.05

Total estimated cost per second-stage plate: $0.13043

Notes:

- PA cost is based on 5 mL of medium per pre-culture tube (each tube inoculates five 9 cm plates) plus 20 mL per first-stage plate, for a total of 21 mL (0.021 L) per second-stage plate.
- Only disposable consumables are included; reusable items (e.g. glass tubes and centrifuge tubes) are excluded.
