# Supplementary material for: A practical guide to two-stage sporulation of Pyricularia oryzae: introducing a filter paper method and comparison with existing methods using strains from diverse grass hosts
Source: Plant Methods. 2025 Nov 18;21:151. doi: 10.1186/s13007-025-01466-6 (PMC12625182; doi:10.1186/s13007-025-01466-6)
Supplement: Supplementary file 3 — SuppFile3_Mycelial_mat_method.pdf – Mycelial mat method. [file 13007_2025_1466_MOESM3_ESM.pdf]

MYCELIAL MAT METHOD

Supplementary Protocol 3: Mycelial mat method (Latterell and Rossi, 1986)

Last edit July 13, 2025

PROTOCOL

Step 1 [Time required 5–6 days]

Pre-culture

1. Inoculate at least 20 finely chopped *P. oryzae* mycelial agar blocks (1–2 mm) into 70 mL of YED broth in a 300 mL Erlenmeyer flask.
2. Incubate on a rotary shaker at 130 rpm at room temperature (approximately 25°C) for 5–6 days (Fig. S1).

> In the original protocol, conidia were used as the inoculum, which promoted the formation of compact mycelial pellets suitable for downstream processing. However, this is not practical for strains that do not sporulate readily under standard conditions. In this protocol, we used finely chopped mycelium instead to accommodate a wider range of strains.

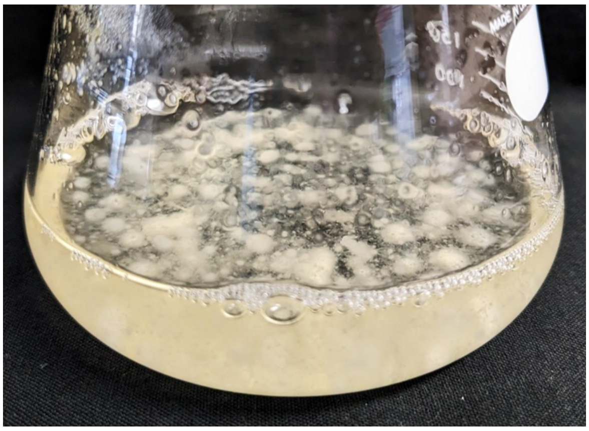

Figure S1: Typical morphology of *P. oryzae* after 5–6 days of cultivation in YED broth.

Step 2 [Time required 3–4 days]

First stage

1. Transfer 35 mL of the pre-culture into 200 mL of sterile YED broth in a 500 mL Erlenmeyer flask (Fig. S2A).
2. Incubate at 130 rpm at room temperature for 3–4 days (Fig. S2B).

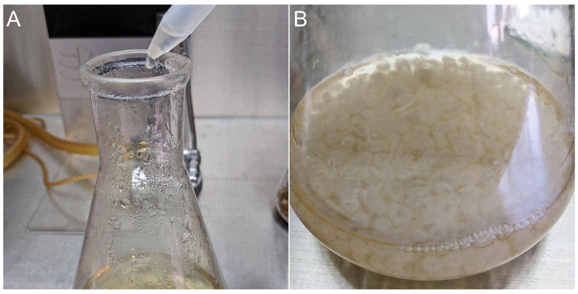

Figure S2: (A) Transfer of pre-culture into fresh YED medium. (B) Typical appearance after 3–4 days of incubation. Pigment production may differ depending on the strain.

MATERIALS

Checklist of required items

Equipment:

- Rotary shaker (set to 130 rpm at room temperature)
- Incubator or temperature-controlled space (maintained at 25 °C)
- Autoclave (for media and tool sterilization)
- Pipettes or sterile droppers
- 300 mL and 500 mL Erlenmeyer flasks with breathable closures (e.g., cotton plugs, foil caps, or vented caps)
- Büchner funnel (9 cm diameter)
- Vacuum pump (for filtration)

Consumables:

- Yeast extract dextrose (YED) medium
  - Yeast extract (3 g/L)
  - Dextrose (15 g/L)
- Filter paper (9 cm diameter, suitable for Büchner funnel)
- Petri dishes (plastic or glass, 9 cm)
- Sterile water

Step 3 [Time required 3–4 days]

Second stage

1. Place a sterile 9 cm filter paper in a Büchner funnel and moisten it with a few drops of sterile water (Fig. S3A, S3B).
2. Turn on the vacuum pump to initiate filtration.
3. Slowly pour the entire 200 mL culture into the funnel (Fig. S3C). After most of the liquid has passed through, rinse the funnel with a small volume of sterile water to wash down residual mycelial fragments (Fig. S3D). Stop suction before the mat becomes overly dry or curls. If needed, rehydrate with 1–2 mL of sterile water.
4. Using sterile forceps, transfer the entire filter paper with the mycelial mat to a sterile 9 cm Petri dish (Fig. S3E). If the mat is too large for the base, place it on the lid, which provides a slightly larger surface (Fig. S3F).
5. Incubate for 3–4 days at 25 °C in the specialized sporulation environment (see Supplementary Protocol 6), under near-UV (365 nm blacklight) or standard fluorescent lighting.

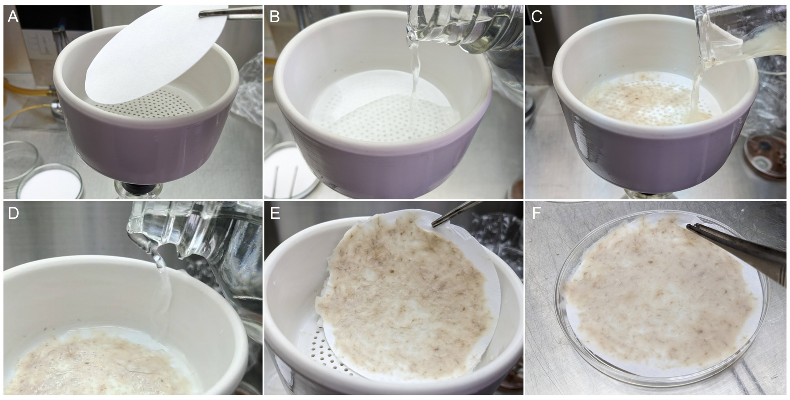

Figure S3: Second-stage procedure. (A) Filter paper placed in a Büchner funnel. (B) Moistened with sterile water. (C) Culture poured into the funnel. (D) Rinsing remaining mycelia. (E) Transferring the mat. (F) Petri dish incubation.

- The original study used a 12 cm Büchner funnel and 400 mL culture. In this version, the procedure was scaled down to use 9 cm filter paper with 200 mL culture.
- To ensure consistency with other protocols and simplify handling, the mycelial mat is incubated on the filter paper inside the Petri dish, rather than being transferred to a separate support such as a wire mesh.

TROUBLESHOOTING

Caution: This method may only be suitable for a subset of *Pyricularia oryzae* strains. If persistent failures occur, consider using an alternative protocol.

- **Low spore yield with little or no aerial mycelium:**
  - Transfer to the second stage before colonies reach the stationary phase; aging mycelium may lose the ability to produce spores efficiently. Try adjusting the first stage incubation in 12 h increments, either reducing or extending as needed.
  - Maintain high humidity ($\geq$97%) by adjusting the number of ventilation holes and keeping the paper towel beneath the dishes moist.
  - Keep the incubation temperature between 25–30 °C.
  - Check for contamination and confirm that the culture remains pure throughout both stages.
- **Low spore yield despite abundant aerial mycelium:**
  - Ensure that the incubation period of first stage is long enough for the culture to reach the late log phase, when sporulation is most efficient. Try increasing the first-stage incubation by 12-h increments.
  - If excessive condensation or a noticeable ammonia odor is observed—both indicators of inadequate aeration—optimize airflow by increasing the ventilation openings in the plastic wrap or decreasing the plate density within the incubator.
  - If failures persist, consider that the strain you are using may not be compatible with this method.
- **High levels of contamination:**
  - Perform all pre-culture and first-stage steps under aseptic conditions. Avoid touching the rim of the flask and Büchner funnel; flame-sterilize all tools and vessel openings before and after use.
  - Ensure that all equipment and any water used for humidification are sterile.
- **Phenotypic changes in colonies:**
  - Use strains purified by mycelial tip or single-spore isolation. Avoid excessive subculturing, especially on nutrient-rich media such as potato dextrose agar.
  - For long-term storage, use filter paper preservation and revive strains from preserved stock only when necessary.
- **Many germinated or empty spores in harvested samples:**
  - Maintain proper humidity during the second stage to prevent condensation on aerial mycelium or conidiophores.
  - Keep incubation under seven days in the second stage to avoid premature germination or over-aging of spores.
  - In high-yield strains, early spore formation may occur on flask walls. Slightly adjust the first-stage agitation speed to reduce mycelial growth on the walls, which can lead to premature sporulation.

COST ANALYSIS

Consumable costs

- Yeast extract: \$0.09/g
- Dextrose: \$0.03/g
- Filter paper (9 cm): \$0.03/piece
- Petri dish (9 cm): \$0.05/piece

Estimated consumable cost per second-stage plate

For each 9 cm plate in the second stage, the estimated consumable costs are:

- **YED medium:** \$0.72/L $\times$ 0.235 L = \$0.1692
- **Filter paper:** \$0.03/piece $\times$ 1 piece = \$0.03
- **Petri dish:** \$0.05/piece $\times$ 1 piece = \$0.05

Total estimated cost per second-stage plate: \$0.2492

Notes:

- YED volume is based on the total used per mat: 35 mL from the pre-culture and 200 mL in the first stage (total 235 mL or 0.235 L).
- Only disposable consumables are included. Reusable items (e.g., Erlenmeyer flasks, Büchner funnel, vacuum pump) are not considered.
