# Supplementary material for: A practical guide to two-stage sporulation of Pyricularia oryzae: introducing a filter paper method and comparison with existing methods using strains from diverse grass hosts
Source: Plant Methods. 2025 Nov 18;21:151. doi: 10.1186/s13007-025-01466-6 (PMC12625182; doi:10.1186/s13007-025-01466-6)
Supplement: Supplementary file 4 — SuppFile4_Corn_grain_method.pdf – Corn grain method. [file 13007_2025_1466_MOESM4_ESM.pdf]

CORN GRAIN METHOD

Supplementary Protocol 4: Corn grain method (Latterell and Rossi, 1986)

Last edit July 13, 2025

PROTOCOL

Step 1 [Time required 5–6 days]

Pre-culture

1. Inoculate at least 20 finely chopped *P. oryzae* mycelial agar blocks (1–2 mm) into 70 mL of YED broth in a 300 mL Erlenmeyer flask.
2. Incubate on a rotary shaker at 130 rpm at room temperature ($\approx$ 25°C) for 5–6 days (Fig. S1).

> Latterell and Rossi (1986) did not specify pre-culture conditions in detail. Here, we applied the same parameters as in their mycelial mat method described in that study.

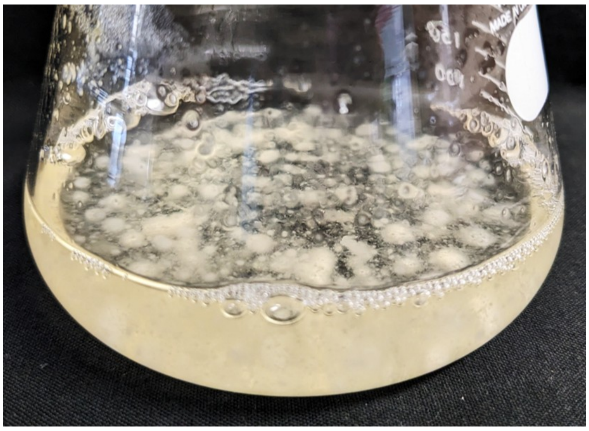

Figure S1: Typical colony morphology of *P. oryzae* after 5–6 days in YED broth.

Step 2 [Time required 5–7 days]

First stage

1. Boil corn grains in a volume of water at least twice their weight for 10 minutes, then let them stand for 30 minutes (Fig. S2A, B).
2. Transfer 100 g of soaked grains into a 300 mL Erlenmeyer flask and seal with a cotton plug wrapped in tissue (Fig. S2D).
3. Autoclave the flask.
4. After cooling, inoculate each flask with 10 mL of the pre−culture suspension using a sterile pipette (Fig. S2E).
5. Incubate the flasks at room temperature until grains are fully colonized, typically 5–7 days (Fig. S2F).

> - Boil grains thoroughly without allowing them to burst; up to 10% rupture is acceptable. Excess rupture produces a sticky substrate and leaches nutrients, affecting sporulation.
> - The dry weight of grains roughly doubles after boiling.
> - Originally, Latterell and Rossi (1986) used 200 g per flask; we scaled this down to 100 g to accommodate more samples.
> - The original method (Latterell and Rossi, 1986) employed active aeration; here, cotton plugs simplify the setup and minimize contamination risk.

MATERIALS

Checklist of required items

Equipment:

- Incubator or temperature-controlled space (25 °C)
- Rotary shaker (set to 130 rpm at room temperature)
- Autoclave (for media and tool sterilization)
- 300 mL Erlenmeyer flask
- Cotton plugs

Consumables:

- Yeast extract dextrose (YED)
  - Yeast extract (3 g/L)
  - Dextrose (15 g/L)
- Dry corn grain
- Petri dishes (plastic or glass, 9 cm)

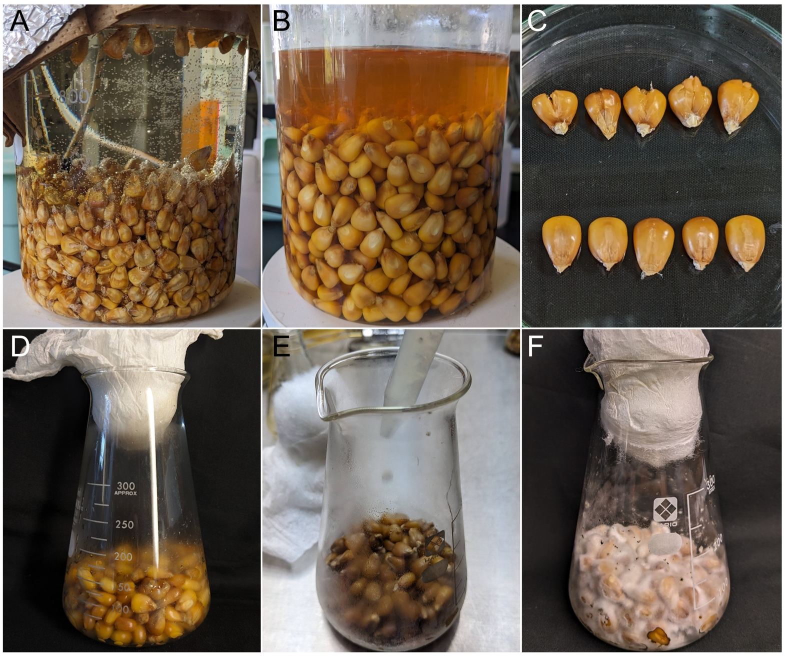

Figure S2: First-stage procedure of the corn grain method. (A, B) Grains boiled and soaked; (C) overcooked grains with ruptured endosperm (top) versus properly cooked grains (bottom); (D) grains loaded into flask with cotton plug; (E) inoculation with 10 mL pre–culture suspension; (F) grains fully colonized after 5–7 days.

Step 3 [Time required 3–4 days]

Second stage

1. Vigorously shake the flask to disaggregate colonized grains (Fig. S3A–B).
2. Transfer approximately 25 g of grains into each sterile 9 cm Petri dish, spreading evenly (Fig. S3C).
3. Incubate for 3–4 days at 25 °C in the specialized sporulation environment (see Supplementary Protocol 6), under near-UV (365 nm blacklight) or standard fluorescent lighting.

- If grains remain clumped by mycelium, separate them with a sterile spoon or scalpel.
- Although Latterell and Rossi (1986) transferred grains onto wire mesh trays, we used 9 cm Petri dishes for direct comparison across methods and found they yield consistent results; one 100 g flask typically produces four dishes.

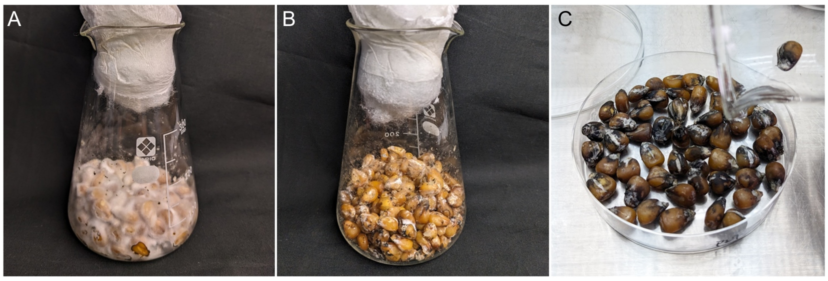

Figure S3: Second-stage procedure of the corn grain method. (A) Grains immediately after second-stage incubation, fully colonized; (B) grains after vigorous shaking; (C) transfer of approximately 25 g of shaken grains into a Petri dish.

TROUBLESHOOTING

- **Low spore yield with little or no aerial mycelium:**
  - Transfer to the second stage before colonies reach the stationary phase; aging mycelium may lose the ability to produce spores efficiently.
  - Maintain high humidity ($\geq$97%) by adjusting the number of ventilation holes and keeping the paper towel beneath the dishes moist.
  - Keep the incubation temperature between 25–30 °C.
  - Check for contamination and confirm that the culture remains pure throughout both stages.
- **Low spore yield despite abundant aerial mycelium:**
  - Ensure that the incubation period of first stage is long enough for the culture to reach the late log phase, when sporulation is most efficient.
  - Avoid overcooking grains during boiling; excessive rupture releases sticky substances that inhibit sporulation.
  - If excessive condensation or a noticeable ammonia odor is observed—both indicators of inadequate aeration—optimize airflow by increasing the ventilation openings in the plastic wrap or decreasing the plate density within the incubator.
- **High levels of contamination:**
  - Perform all pre-culture and first-stage steps under aseptic conditions. Avoid touching the rim of the flask. If cotton plugs become wet during sterilization, dry them in a sterile environment before use or replace with fresh sterile cotton. Flame-sterilize tools and vessel openings before and after use.
  - For environments prone to mite infestation, cotton plugs may not suffice; consider using silicone breathable lids fitted with PTFE or silica membrane filters.
  - Ensure that all equipment and any water used for humidification are sterile.
- **Phenotypic changes in colonies:**
  - Use strains purified by mycelial tip or single-spore isolation. Avoid excessive subculturing, especially on nutrient-rich media such as potato dextrose agar.
  - For long-term storage, use filter paper preservation and revive strains from preserved stock only when necessary.
- **Many germinated or empty spores in harvested samples:**
  - Maintain proper humidity during the second stage to prevent condensation on aerial mycelium or conidiophores.
  - Keep incubation under seven days in the second stage to avoid premature germination or over-aging of spores.
  - In high-yield strains, some spores may form during the first stage and subsequently germinate or become empty during the second stage. To remove pre-existing spores, rinse grains with sterile water several times before plating.

COST ANALYSIS

Consumable costs

- Yeast extract: \$0.09/g
- Dextrose: \$0.03/g
- Corn grain (dry): \$0.00/g
- Petri dish (9 cm): \$0.05/piece

Estimated consumable cost per second-stage plate

For each 9 cm plate in the second stage of the corn grain method, the costs are:

- **YED medium:** \$0.72/L$\times$0.0025 L = \$0.0018
- **Corn grain:** \$0.005/g$\times$12.5 g = \$0.0625
- **Petri dish:** \$0.05/piece $\times$ 1 piece = \$0.05

Total estimated cost per second-stage plate: \$0.1143

Notes:

- The volume of YED (2.5 mL or 0.0025 L) is derived from a preculture inoculum 10 mL distributed over four plates of the second stage.
- The dry weight 12.5 g of corn grain corresponds to the wet weight 25 g per plate, given that boiling roughly doubles the grain weight.
- Only disposable consumables are included; reusable items (e.g. Erlenmeyer flask, cotton plug) are excluded.
