# Supplementary material for: A practical guide to two-stage sporulation of Pyricularia oryzae: introducing a filter paper method and comparison with existing methods using strains from diverse grass hosts
Source: Plant Methods. 2025 Nov 18;21:151. doi: 10.1186/s13007-025-01466-6 (PMC12625182; doi:10.1186/s13007-025-01466-6)
Supplement: Supplementary file 5 — SuppFile5_TARI_method.pdf – TARI method [file 13007_2025_1466_MOESM5_ESM.pdf]

# TARI METHOD

## Supplementary Protocol 5: TARI method (Chen et al. 2021)

Last edit July 13, 2025

## PROTOCOL

Step 1 [Time required 4 days]

### Pre-culture

1. Inoculate the *P. oryzae* strain onto V8A juice agar, using one or two points of agar plug.
2. Incubate at room temperature (≈25 °C) in the dark for 4 days (Fig. S1).

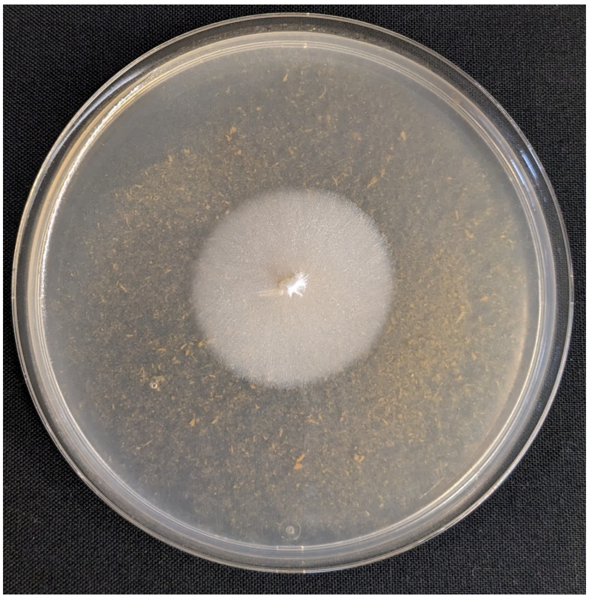

Figure S1: Pre-culture of *P. oryzae* on V8A after 4 days. Colony color and size vary among strains.

---

Step 2 [Time required 4 days]

### First stage

1. Use a sterile scalpel to cut the 4-day-old colony into small fragments, then transfer into 50 mL of PB in a 125 mL flask (Fig. S2A-B).
2. Incubate in complete darkness at 28 °C with shaking at 130 rpm for 4 days (Fig. S2C-D).

- The composition of PB differs from that of PA used in the IRRI method.
- Fungal liquid cultures remain clear even if some strains produce dark pigment; any cloudiness or unusual odor indicates bacterial contamination.

## MATERIALS

### Checklist of required items

**Equipment:**

- Incubator shaker (25 °C, 130 rpm)
- Blender (Ensure the blender jar and blades are autoclavable)
- Autoclave (for media and tool sterilization)
- Pipettes or droppers
- 125 mL and 300 mL Erlenmeyer flasks
- Triangle spreader

**Consumables:**

- V8 juice agar (V8A):
  - V8 juice, 100 mL/L
  - $CaCO_3$, 0.2 g/L
  - Agar, 17 g/L
- Prune broth (PB):
  - Prunes, 15 g/L (≈1.5 pieces)
  - Starch, 2.5 g/L
  - Yeast extract, 0.5 g/L
  - Adjust pH to 6.5
- Oat flour agar (OFA):
  - Oat flour, 10 g/L (finely ground)
  - Agar, 17 g/L
- Petri dishes (plastic or glass, 9 cm)

- To prepare Prune Broth (PB): boil prunes in half the final volume of water for 1 h, crush and sieve out solids, then adjust to the final volume and add remaining ingredients. Adjust pH to 6.5 using NaOH before heating to avoid agar degradation. If a pH meter is unavailable, 0.1 % $CaCO_3$ may be used to buffer the medium.
- Oat flour is obtained by grinding commercial rolled oats to a fine powder.

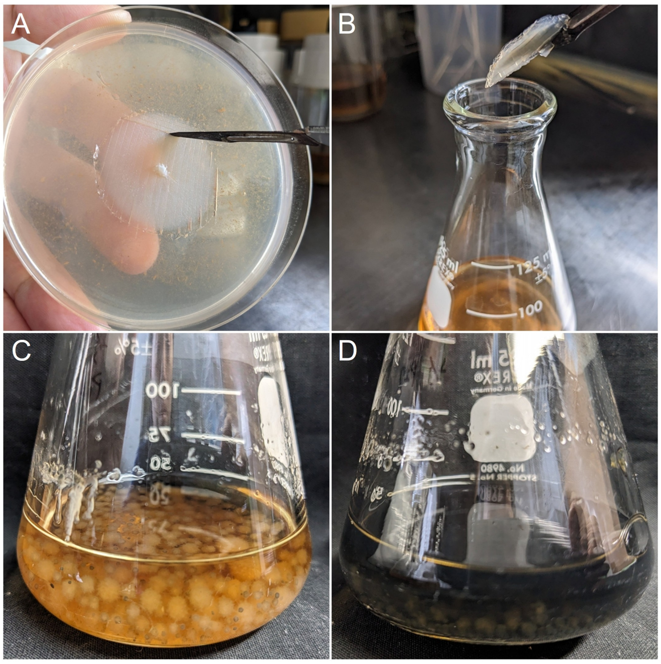


Figure S2: First-stage TARI method. (A) Cut the 4-day-old colony into small fragments. (B) Fragments transferred to PB. (C–D) Typical appearance after 4 days: white pellets (C) or pronounced dark pigment (D) depending on the strain.

Step 3 [Time required 3–4 days]

### Second stage

1. Transfer the entire first-stage culture, including mycelium, into a sterile blender (Fig. S3A) and blend on low speed for 15 s (Fig. S3B).
2. Using a sterile dropper, dispense 2 mL of the blended suspension onto each 9 cm OFA plate (Fig. S3C).
3. Spread the suspension evenly with a sterile spreader and air-dry under a clean bench until no visible moisture remains (Fig. S3D–E).
4. Incubate for 3–4 days at 25 °C in the specialized sporulation environment (see Supplementary Protocol 6), under near-UV (365 nm blacklight) or standard fluorescent lighting.

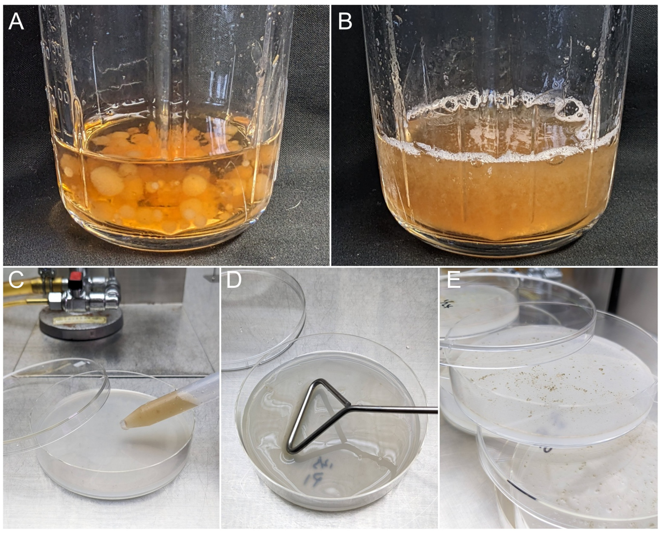


Figure S3: Second-stage TARI method. (A) Culture before blending. (B) Blended suspension. (C) Inoculation of blended suspension on OFA. (D) Spreading suspension with a sterile spreader. (E) Air-drying under a clean bench.

# TROUBLESHOOTING

- **Low spore yield with little or no aerial mycelium:**
  - Transfer to the second stage before colonies reach the stationary phase; aging mycelium may lose the ability to produce spores efficiently. For strains that begin pigment production before day 4 of the first stage, proceeding to the second stage on the day pigment appears can sometimes improve yield.
  - Maintain high humidity ($\geq$97%) by adjusting the number of ventilation holes and keeping the paper towel beneath the dishes moist.
  - Keep the incubation temperature between 25–30 °C.
  - Check for contamination and confirm that the culture remains pure throughout both stages.
- **Low spore yield despite abundant aerial mycelium:**
  - Ensure that the incubation period of first stage is long enough for the culture to reach the late log phase, when sporulation is most efficient.
  - After sterilizing OFA, gently swirl the container before and during dispensing to keep oat flour evenly suspended, preventing sediment build-up and nutrient-rich spots that inhibit sporulation.
  - If excessive condensation or a noticeable ammonia odor is observed—both indicators of inadequate aeration—optimize airflow by increasing the ventilation openings in the plastic wrap or decreasing the plate density within the incubator.
- **High levels of contamination:**
  - Perform all pre-culture and first-stage steps under aseptic conditions. Avoid touching the rim of the blender jar directly; flame-sterilize all tools and vessel openings before and after use.
  - Ensure that all equipment and any water used for humidification are sterile.
- **Phenotypic changes in colonies:**
  - Use strains purified by mycelial tip or single-spore isolation. Avoid excessive subculturing, especially on nutrient-rich media such as potato dextrose agar.
  - For long-term storage, use filter paper preservation and revive strains from preserved stock only when necessary.
- **Many germinated or empty spores in harvested samples:**
  - Maintain proper humidity during the second stage to prevent condensation on aerial mycelium or conidiophores.
  - Keep incubation under seven days in the second stage to avoid premature germination or over-aging of spores.
  - In high-yield strains, spores may form on mycelium that sticks to the flask walls during the first stage. To prevent this, fine-tune the shaker speed or gently tap the flasks periodically to keep the mycelium in suspension.

# COST ANALYSIS

## Consumable costs

- V8 juice: $0.02/mL
- $CaCO_3$: $0.04/g
- Agar: $0.17/g
- Prunes: $0.15/piece
- Starch: $0.028/g
- Yeast extract: $0.09/g
- Oat flour: $0.003/g
- Petri dish (9 cm): $0.05/piece

## Estimated consumable cost per second-stage plate

For each 9 cm OFA plate in the second stage, the estimated costs are:

- **V8 juice agar (V8A):** $4.898/L× 0.0008 L = $0.003918
- **Prune broth (PB):** $0.34/L× 0.002 L = $0.00068
- **Oat flour agar (OFA):** $2.92/L× 0.02 L = $0.0584
- **Petri dish:** $0.05/piece × 1.04 piece = $0.052

**Total estimated cost per second-stage plate: $0.1150**

*Notes:*

- V8A usage is based on one 50 mL pre-culture yielding 25 second-stage plates; each plate requires 20 mL/25 = 0.8 mL (0.0008 L) of V8A.
- Petri dish cost accounts for 1/25 dish per pre-culture plus 1 dish per second stage, totaling 1.04 dishes per plate.
- Only disposable consumables are included; reusable items (e.g., blender jar, flasks) are excluded.
