# Supplementary material for: A practical guide to two-stage sporulation of Pyricularia oryzae: introducing a filter paper method and comparison with existing methods using strains from diverse grass hosts
Source: Plant Methods. 2025 Nov 18;21:151. doi: 10.1186/s13007-025-01466-6 (PMC12625182; doi:10.1186/s13007-025-01466-6)
Supplement: Supplementary file 6 — SuppFile6_Setup_of_Sporulation_Environment.pdf – Setup of the sporulation environment. [file 13007_2025_1466_MOESM6_ESM.pdf]

SETUP OF SPORULATION ENVIRONMENT

Supplementary Protocol 6: Setup of sporulation environment

Last edit October 14, 2025

PROTOCOL

Step 1 [Time required 3–4 days]

Second-Stage Incubation Environment

1. Line a sterile tray (30×20×5 cm) with autoclaved, damp paper towels to sustain $\geq$97% relative humidity (Fig. S1A).
2. Place second-stage culture plates in a single, non-overlapping layer on the damp towels (Fig. S1A).
3. Cover the tray with plastic wrap and secure with clips. Using sterile tweezers, create multiple small perforations (1–2 mm diameter) to allow air exchange while retaining humidity (Fig. S1B–C).
4. Incubate at 25 °C under near-UV (365 nm blacklight) or standard fluorescent lighting for 3–4 days (Fig. S1D).

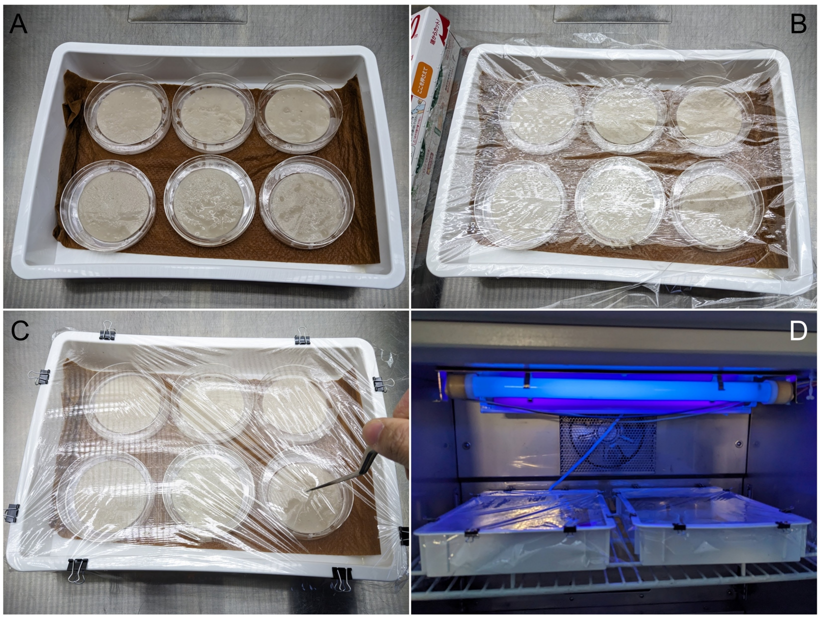

Figure S1: Second-stage incubation setup: (A) Culture plates arranged on damp paper towels; (B) Tray sealed with plastic wrap; (C) Small perforations allow air exchange while retaining humidity; (D) Incubation under blacklight.

MATERIALS

Checklist of required items

Equipment:

- Incubator or temperature-controlled space (25 °C)
- Near-UV light source (365 nm blacklight tube or LED); standard fluorescent lighting acceptable if unavailable
- Clips for sealing trays
- Sterile tweezers or a puncturing tool for ventilation holes

Consumables:

- Plastic wrap
- Autoclaved paper towels
- Sterile water (for moistening paper towels)

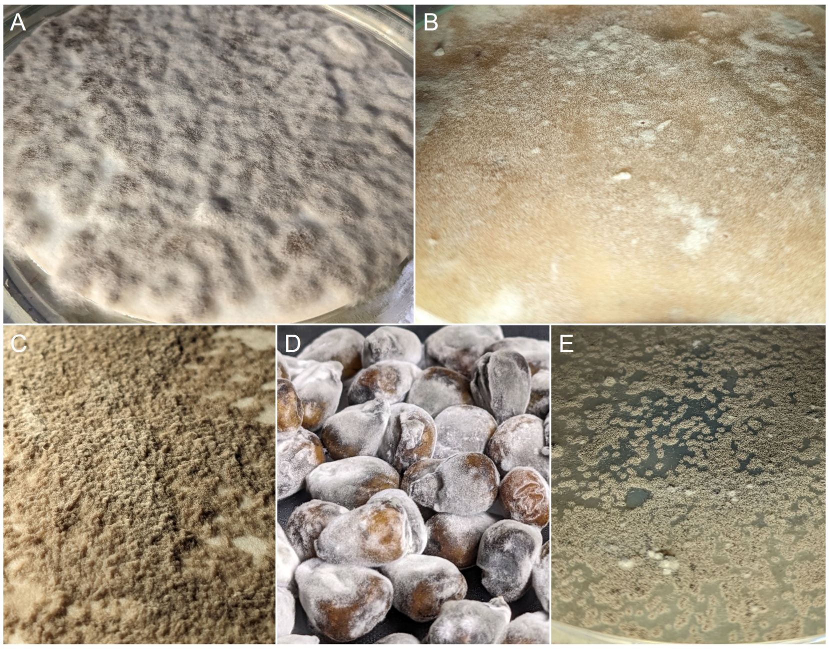

Figure S2: Typical sporulation patterns on substrates after second-stage incubation: (A) Filter paper method; (B) IRRI method; (C) Mycelial mat method; (D) Corn grain method; (E) TARI method.

- Adjust hole number and size to balance humidity retention and ventilation; slight substrate surface drying by day 4 is normal and helps prevent premature spore germination.
- Near ultraviolet light (365 nm) promotes sporulation. Do not use the germicidal UVC lamp (254 nm) found in clean benches or biosafety cabinets; it will kill the fungus. If a near ultraviolet source is not available, standard fluorescent lighting is an acceptable alternative.
- If using a humidity-controlled incubator, regularly inspect and disinfect to avoid contamination from condensate.
