# Supplementary material for: A practical guide to two-stage sporulation of Pyricularia oryzae: introducing a filter paper method and comparison with existing methods using strains from diverse grass hosts
Source: Plant Methods. 2025 Nov 18;21:151. doi: 10.1186/s13007-025-01466-6 (PMC12625182; doi:10.1186/s13007-025-01466-6)
Supplement: Supplementary file 7 — SuppFile7_Strain_list.pdf – Strains used in this study and in the phylogenetic analysis. [file 13007_2025_1466_MOESM7_ESM.docx]

Table S1. Strains used in this study, including alternative names, host or source, locality, and year of collection.

| Strain name  used in this study | Other strain names^a^ | Host | Locality | Genbank accession No. |
| --- | --- | --- | --- | --- |
| p1 | MAFF 511695, NIR2021-3 | *Lolium multiflorum* | Japan | **LC876988** |
| p4 |  | *Lolium multiflorum* | Japan | **LC876989** |
| p7 |  | *Lolium perenne* | Japan | **LC876991** |
| p8 | MAFF 101101 | *Lolium multiflorum* | Japan | **LC876994** |
| p11 | MAFF 239329 | *Echinochloa* sp. | Japan | **LC876997** |
| p12 | MAFF 239346 | *Lolium multiflorum* | Japan | **LC876992** |
| p13 | MAFF 240215 | *Eleusine coracana* | Japan | **LC876996** |
| p14 | MAFF 240216 | *Eleusine coracana* | Japan | **LC876998** |
| p17 | MAFF 305510 | *Lolium multiflorum* | Japan | **LC876999** |
| p18 | MAFF 305527 | *Zea mays* | Japan | **LC877003** |
| p27 | naga-1 | *Oryza sativa* | Japan | **LC877004** |
| p29 | SET1 | *Setaria viridis* | Japan | **LC877006** |
| p30 | THWERy1 | *Lolium × boucheanum* | Japan | **LC876993** |
| p31 | THWEDI1 | *Digitaria ciliaris* | Japan | **LC877009** |
| p32 | Aki1 | *Digitaria ciliaris* | Japan | **LC877010** |
| p47 | MAFF 101023 | *Setaria italica* | Japan | **LC877007** |
| p48 | MAFF 101024 | *Setaria italica* | Japan | **LC877008** |
| p49 | MAFF 239335 | *Lolium pratense* | Japan | **LC877005** |
| p50 | MAFF 239348 | *Lolium arundinaceum* | Japan | **LC876990** |
| p51 | MAFF 243103 | *Avena strigosa* | Japan | **LC876995** |
| p3t | TJ4a3 | *Oryza sativa* | Taiwan | **LC877000** |
| p4t | YL2a3 | *Oryza sativa* | Taiwan | **LC877001** |
| p5t | PT5a5 | *Oryza sativa* | Taiwan | **LC877002** |
| CBS 138707 |  | *Digitaria* sp. | USA | KM484885 |
| GE3 |  | *Digitaria* sp. | Germany | ERR9866257 |
| K23/123 |  | *Eleusine coracana* | Kenya | PHFK01000015 |
| Br62 |  | *Eleusine coracana* | Brazil | CAUVZL010000010 |
| E34 |  | *Eleusine coracana* | Ethiopia | VIDS01000002 |
| MZ5-1-6 |  | *Eleusine coracana* | Japan | GCA_004346965 |
| CD156 |  | *Eleusine indica* | Ivory Coast | UELZ03000010 |
| Lh88405 |  | *Leersia hexandra* | Philippines | GCA_012654035 |
| TF05-1MC7 |  | *Lolium arundinaceum* | USA | JAVBIT010000001 |
| LPKY97 |  | *Lolium perenne* | USA | CP050920 |
| KEN54-20 |  | *Oryza sativa* | Japan | JAODUQ010000037 |
| AG038 |  | *Oryza sativa* | Italy | CAJHIC020000008 |
| Guy11 |  | *Oryza sativa* | Guyana | MQOP01000037 |
| P131 |  | *Oryza sativa* | Japan | GCA_000292605 |
| EA18 |  | *Oryza sativa* | China | GCA_021764705 |
| O-219 |  | *Oryza sativa* | Ivory Coast | GCA_043231905 |
| O-137 |  | *Oryza sativa* | China | GCA_043231915 |
| Rep4-buf1#13 |  | *Oryza sativa* | USA | GCA_025473255 |
| Rep1-buf1#10 |  | *Oryza sativa* | USA | GCA_025473295 |
| US71 |  | *Setaria italica* | USA | UCNY03000020 |
| Arcadia2 |  | *Setaria viridis* | USA | GCA_012654115 |
| ZM2-1 |  | *Triticum aestivum* | Zambia | JAMXQW010000001 |
| Br48 |  | *Triticum aestivum* | Brazil | GCA_036493215 |
| Py22.1 |  | *Triticum aestivum* | Brazil | GCA_002218425 |
| T3 |  | *Triticum aestivum* | Brazil | GCA_030718865 |
| B71 |  | *Triticum aestivum* | Bolivia | GCA_004785725 |
| Br58 |  | *Avena sativa* | Brazil | DRX403521 |
| Ei9411 |  | *Eleusine indica* | China | GCA_001548775 |
| JS-464 |  | *Phragmites communis* | South Korea | GCA_002197995 |
| ^a^ MAFF denotes strain deposit numbers registered in the NARO Genebank, Japan. | | |  |  |
